# Expanding the genetic and clinical spectrum of osteogenesis imperfecta: identification of novel rare pathogenic variants in type I collagen-encoding genes

**DOI:** 10.3389/fendo.2023.1254695

**Published:** 2023-10-20

**Authors:** Francesco Paduano, Rita Fischetto, Biagio Moretti, Danila De Vito, Marco Tatullo

**Affiliations:** ^1^ Stem Cells and Medical Genetics Units, Tecnologica Research Institute and Marrelli Health, Crotone, Italy; ^2^ Metabolic and Genetic Diseases Unit, “Giovanni XXIII” Hospital, Bari, Italy; ^3^ Orthopaedic and Traumathogic Unit General Hospital Policlinico, Department of Translational Biomedicine and Neuroscience, University “Aldo Moro” of Bari, Bari, Italy; ^4^ Department of Translational Biomedicine and Neuroscience, Medical School, University ”Aldo Moro” of Bari, Bari, Italy

**Keywords:** osteogenesis imperfecta (OI), autosomal recessive OI, next generation sequencing (NGS), collagen type one, molecular diagnosis of OI

## Abstract

**Introduction:**

Osteogenesis imperfecta (OI) is a clinically and genetically heterogeneous skeletal disorder. The majority of affected cases are attributed to autosomal dominant pathogenic variants (PVs) found in the *COL1A1* and *COL1A2* genes, which encode type I collagen. However, PVs in other genes involved in collagen posttranslational modification, processing, crosslinking, osteoblast differentiation, and bone mineralization have also been associated with OI.

**Methods:**

In this study, we present the results of next-generation sequencing (NGS) analysis using a custom panel of 11 genes known to be associated with OI. This clinical study enrolled a total of 10 patients, comprising 7 male and 3 female patients from 7 families, all from the Puglia Region in South Italy, providing a detailed overview of their age, gender, family history, OI type, and non-skeletal features.

**Results:**

The genetic analysis revealed 5 PVs in the *COL1A1* gene and 2 PVs in the *COL1A2* gene. Importantly, three of these PVs have not been previously reported in the literature. These include two novel heterozygous frameshift PVs in *COL1A1* (c.2890_2893del and c.3887del) and one novel heterozygous missense PV in *COL1A2* (c.596G>T).

**Discussion:**

The identification of these previously unreported PVs expands the variant spectrum of the *COL1A1* and *COL1A2* genes and may have implications for accurate diagnosis, genetic counselling, and potential therapeutic interventions in affected individuals and their families.

## Introduction

Osteogenesis imperfecta (OI) is a rare genetic disorder characterized by bone fragility and increased susceptibility to fractures. Typical extraskeletal manifestations associated with the disorder include blue sclera, dentinogenesis imperfecta, hyperlaxity of ligaments and skin, hearing impairment, and the presence of Wormian bones on skull radiographs (1). OI represents a clinically and genetically heterogeneous disorder, exhibiting a wide range of inheritance patterns, including autosomal dominant, autosomal recessive, and X-linked modes of transmission (2). The disease displays significant variability in terms of disease severity and associated features (1, 3, 4).

Among the different OI types, Type I is the most prevalent and is characterized by blue sclera and a range of fractures, typically without bone deformities (5). Conversely, OI Type II manifests as a prenatal or perinatal lethal condition, displaying severe deformities, congenital fractures, and a marked deficiency of ossification. Non-lethal cases of OI are often diagnosed as Type III, which represents the most severe form. Individuals with Type III exhibit very short stature, long bone deformities, and multiple spinal issues. Lastly, Type IV of OI is classified as a moderately deforming variant, with phenotypic expression varying in severity. This type is characterized by the presence of multiple fractures and the notable appearance of white or gray sclera (5). The various OI types are intricately tied to specific genetic mutations. Type I, with its non-deforming nature, is linked to mutations in *COL1A1* and *COL1A2* genes. Type II’s severe perinatal form is associated with mutations in *COL1A1*, *COL1A2*, *CRTAP*, *LEPRE1*, *PPIB*, and *BMP1* genes. Type III, progressively deforming, involves mutations in *COL1A1*, *COL1A2*, and additional genes like *FKBP10* and *WNT1*. Type IV, a moderate form, is attributed to genes including *COL1A1*, *COL1A2*, *FKBP10*, *SP7*, and others. The OI Type V characterized by the presence of membrane calcification originates from mutations within the IFITM5 gene. Understanding these genotype–phenotype links offers valuable insights for diagnosis and potential targeted treatments in OI patients (6).

OI affects approximately 1 in 15,000 to 20,000 individuals worldwide, making it one of the most common inherited skeletal dysplasias (7). The disorder is primarily caused by structural pathogenic variants (PVs) in the genes encoding collagen type I, alpha-1 (*COL1A1*), and collagen type I, alpha-2 *(COL1A2)*, which account for 85%–90% of OI cases (8). Modifications in the structure of collagen type I molecules have profound implications across a wide range of tissues, including dermis, cartilage, dentine, sclera, cornea, ligament, tendon, and bone, leading to diverse skeletal and extraskeletal manifestations (9).

Type I collagen has a helical structure built by two alpha-1 protein strands and one alpha-2 protein strand encoded by *COL1A1* and *COL1A2*, respectively. These genes have complex sequencing requirements due to their high number of exons. With 52 exons each, they do not exhibit specific mutation hotspots. As a result, the OI Variant Database lists approximately 2,500 different PVs in *COL1A1* and *COL1A2* (10). The predominant sequence abnormality in OI involves a point mutation impacting a glycine residue in COL1A1 or COL1A2, resulting in the production of a combination of normal and abnormal collagen. The severity of the resulting phenotype ranges from mild to lethal, depending on the affected chain, the location of the substitution within the triple helix, and the specific amino acid replacing glycine (1).

Currently, advancements in genomic technologies have unveiled an expanding genetic landscape of OI, with the identification of at least 16 other genes associated with OI than *COL1A1* and *COL1A2*, including genes involved in collagen synthesis, posttranslational modification, processing, crosslinking, osteoblast differentiation, and bone mineralization (3, 11–13). The large number of genes involved in the pathogenesis of OI are FK506-binding protein 10 (*FKBP10*), peptidyl-prolyl isomerase B (*PPIB*), serpin peptidase inhibitor, clade f, member 1 (*SERPINF1*), wingless-type MMTV integration site family, member 1 (*WNT1*), bone morphogenetic protein 1 *(BMP1)*, cartilage-associated protein *(CRTAP)*, prolyl 3-hydroxylase 1 (*P3H1)*, serpin peptidase inhibitor, clade h, member 1 (*SERPINH1)*, and interferon-induced transmembrane protein 5 (*IFITM5)* (3, 4, 14–16). The identification of rare PVs in genes beyond *COL1A1* and *COL1A2* has challenged the traditional view of OI as a monogenic disorder and has led to a better understanding of the underlying molecular mechanisms and clinical heterogeneity (3, 4). For instance, mutations in *FKBP10* have been shown to disrupt collagen folding and lead to abnormal bone formation (14). Similarly, PVs in *WNT1* have been implicated in impaired osteoblast differentiation and bone mineralization (17). The identification and characterization of PVs in these genes have significantly expanded our understanding of the genetic and molecular basis of OI (4).

In this study, our objective is to broaden the understanding of the genetic and clinical characteristics of OI by investigating rare PVs not only within the well-established *COL1A1* and *COL1A2*, which are responsible for more than 85%–90% of all cases but also in other genes involved in OI. To achieve this, we performed next-generation sequencing (NGS) analysis on OI patients from the Puglia Region in South Italy, a population with limited genetic data on OI. By utilizing a targeted gene panel, including *COL1A1*, *COL1A2*, *FKBP10*, *PPIB*, *SERPINF1*, *WNT1*, *BMP1*, *CRTAP*, *P3H1*, *SERPINH1*, and *IFITM5*, we comprehensively analyzed the coding regions and splice sites of these genes in our patient cohort. We also collected detailed clinical information, such as age, gender, family history, OI type, and non-skeletal features, to facilitate genotype–phenotype correlation.

The genetic analysis of this study, which goes beyond the well-established *COL1A1* and *COL1A2* genes, could be helpful in understanding the genetic and clinical aspects of OI and identifying novel PVs not previously characterized in OI patients.

## Subjects and methods

### Subjects

This study included all individuals with a typical OI phenotype who were evaluated in the Metabolic and Genetic Diseases Unit of the Hospital “Giovanni XXIII”, General Hospital Policlinico, Bari, Italy, between 2013 and 2023. This study was approved by the Institutional Review Board of the General Hospital Policlinico. Written informed consent was obtained from all patients aged 16 years or older whereas written parental consent was obtained for individuals under 16 years of age.

### Clinical evaluation

Clinical information, including medical history, age at diagnosis, gender, family history, and non-skeletal features such as hearing loss, was collected.

### Biological samples and DNA isolation

Blood samples were isolated from 10 patients affected by OI. DNA was isolated from 200 μL of peripheral blood using InviMag Blood DNA mini kit/IG kit and the automated platform for nucleic acid purification InviGenius Plus according to the manufacturer’s instructions (Stratec Molecular GmbH, Berlin, Germany). The concentration of the isolated DNA was determined by Qubit dsDNA HS Assay Kit (ThermoFisher Scientific, Waltham, MA, USA).

### Library preparation and Illumina sequencing

To identify PVs in genes involved in OI, we used a custom panel (Ampliseq custom DNA Panel) designed using the Designstudio V5.0 software (Illumina), which covers 99.93% of the *COL1A1, COL1A2, FKBP10, IFITM5, PPIB, SERPINF1, WNT1, BMP1, CRTAP, P3H1*, and *SERPINH1.* The custom panel consists of 266 total amplicons (1st pool of 133 and 2nd pool of 133 amplicons) having a complete coverage (99.93%) of all genes listed above. For each gene, all exons and adjacent intronic regions ± 25 nucleotides were sequenced (exon padding: 25). The only bases that are not covered by the *in silico* design were 22 bases of the padding (intron) of exon 8 of the *FKBP10* gene, due to the presence of a highly repeated region (homopolymer region).

Briefly, DNA samples were diluted to 10 ng/μL, and subsequently, the target regions were amplified by PCR using the Ampliseq kit (Illumina) and the custom panel containing the specific primers of the genes listed above. The generated amplicons were partially digested with the FuPa reagent and then the adapter (i5/i7) indexes (Ampliseq UD indexes, Illumina) necessary for sequencing were added by PCR. A clean-up of the libraries was then carried out with the AMPure XP magnetic beads with a subsequent amplification of the libraries to ensure an adequate quantity of sequences for the Illumina system. In the next phase, a second clean-up has been performed and then all libraries were quantified by the QUBIT fluorometer (Invitrogen). All the generated libraries were checked on the bioanalyzer 2100 (Agilent) to evaluate their quality before NGS sequencing. As expected, each library presents a correct profile of the amplicons with an average size of approximately 390 bp.

### NGS sequencing

In the final step, each library was diluted to 10 nM in Low TE, and then all libraries were pooled at 10 nM and diluted to 4 nM. Finally, the pooled libraries were denatured and diluted at 12 pM and loaded on a MiSeq Reagent Nano Kit v2 (300-cycles) cartridge with 12.5 pM *PhiX* (1.5%) sequencing control V3 (Illumina). Libraries were then sequenced on Illumina MiseqDX (Illumina) instrumentation in RUO mode. The whole process also required the use of a Veriti 96 thermal cycler (Applied Biosystem) for the amplification of the libraries and the Dynamag-96 side (Invitrogen) for the clean-up of the libraries.

### Evaluation of NGS performance

NGS performance was evaluated using the DNA Amplicon module (Illumina). The qualitative analysis of the NGS run shows optimal metrics that have passed all the quality controls of the primary analysis including %Q30>84.02, a percentage (%) of PF (Passing Filter) equal to 94.07% with a yield of approximately 442 Mb. The cluster density is also in specification with 1.091 K. Furthermore, the percentage of *PhiX* loaded turns out to be 1.36% with an error rate of 0.47%. The secondary analysis of the NGS run for each sample in terms of coverage and uniformity results in optimal values with an average Amplicon coverage greater than 1,000 and a uniformity greater than 96%. Typically, the average coverage of 100–300 with a coverage (uniformity of coverage) of 90% of the amplicons is sufficient for an NGS analysis of germ samples. In our analyses, we obtained an average coverage (Amplicon mean coverage) greater than 1,000 and uniformity of coverage greater than 96% for each sample; these are optimal values to proceed with a correct tertiary analysis (variants of biological significance/germline variants).

### Variant calling

Data (VCF files) were obtained using the DNA Amplicon (Illumina) module and analyzed by Evai (Engenome, https://evai.engenome.com/#app/analysis/11919), Variant Interpreter (Illumina, https://variantinterpreter.informatics.illumina.com/registry/cases), and Varsome Clinical (https://varsome.com). Briefly, the DNA Amplicon module was used to align the reads to the reference genome (hg19) and then to run to search for germline variants in the targeted regions. Variant classification is based on the current ACMG standards and guidelines. All variants were reviewed using VARSOME (https://varsome.com), the Osteogenesis Imperfecta Variant Database (oi.gene.le.ac.uk), dbSNP (http://www.ncbi.nlm.nih.gov/projects/SNP/), and Clinvar (https://www.ncbi.nlm.nih.gov/clinvar/).

The software VarSeq 2.3.0 (Golden Helix) was also used to confirm PVs detected in this study.

## Results

### Clinical features of OI patients and family pedigree

In this study, we enrolled 10 patients from seven different families, comprising 7 male and 3 female patients. The age range was between 4 and 57 years, and the cohort included six pediatric (<18 years) and four adult patients. Clinical information, including age, gender, family history, OI type, and non-skeletal features like blue sclera and hearing loss, and other clinical details were collected (**Table 1**). In addition, X-ray radiography of the upper and lower extremities was examined for patients 22/21 and 02/22 (**Supplementary Image 1, 2**).

**Table 1:** Clinical features of the patient cohort.

Patient	Internal code	Gender	Age (years)	Family number and family history	OI type	Blu sclera	Hearing loss	Supplementary clinical details
1	21/21	Male	18	Family 1 Mother has OI	1	Yes	No	The disease was diagnosed at birth. The patient has undergone a complete spinal X-ray, and bone densitometry (DEXA) with a Z-score of −2.2. At the last admission, the patient’s stature age was 13 years and 7 months, weight age was 15 years and 5 months, and BMI was 23.167. Two fractures were reported, affecting the head of the olecranon, first on the right and then on the left, due to accidental falls of small entities and an impact with a metal structure.
2	22/21	Male	6	Family 2 Father (Patient 23/21), Uncle, and Grandmother have OI	1	Yes	No	Reflux surgery was reported. Two tibial fractures were reported at the age of 12 months due to minor falls. A delay in language acquisition was reported. Following an orthopedic consultation, the patient was found to have pronated flat feet. The spine is in alignment without axial deviations or gibbosity, and the limbs are in alignment. An otolaryngological consultation found that the patient had grade II tonsils, with a normal rhinoscopic examination. Infant audiometric testing was within normal limits. Dental consultation revealed that deciduous dentition was completed according to age and sex. No dental lesions were visible that could be attributed to congenital conditions. A nephrological consultation found that the child with OI had undergone reflux surgery. Renal ultrasound revealed hyper-echogenic spots in the calyx and parenchyma bilaterally, more evident on the right side (right 3 mm); right pyelectasia 6 mm with thickening of the pyelic wall, distal ureters visible (right 7 mm, left 5 mm).
3	23/21	Male	42	Family 2 Son (Patient 22/21), Mother, and Brother have OI	1	Yes	No	No supplementary clinical details
4	24/21	Male	15	Family 3 Mother has OI (Patient 25/21)	1	Yes	Yes	Born at term by emergency Cesarean section for failure to engage. Normal pregnancy. At birth, the proband had a birth weight of 3.6 kg. Perinatality and the first stages of psychomotor development are reported as normal. Twelve fractures at the age of 1 year for which he was hospitalized with a diagnosis of Osteogenesis Imperfecta type I. Diagnosis of dysgraphia and dysarthrography, for which he undergoes follow-up. Previous thyroid ultrasound shows the thyroid in place, morpho dimensionally within normal limits, with homogeneous echostructure devoid of evident nodular lesions. As usual, the vascular signal is at PWD evaluation. Trachea in axis (dimensions reported on the left side of the echograms). The patient suffered a nearly composed fracture at the distal diaphyseal third of the tibia and presents diffuse osteopenia. The patient presents over the years a series of fractures; composed fracture third distal tibia dx, a fracture of the proximal phalanx of the V finger, near which, on the internal side, a minute calcification is reported in the context of subcutaneous soft tissues.
5	25/21	Female	48	Family 3 Son has OI (Patient 24/21)	1	Yes	Yes	No supplementary clinical details
6	01/22	Female	14	Family 6: No affected family members	1	Yes	No	An abdominal ultrasound performed at 6 years of age revealed that the liver was within normal size and had a regular echostructure. The gallbladder did not have any stones, and the biliary tract was not dilated. The portal vein appeared regular, and the pancreas and spleen were within normal limits.
7	02/22	Female	4	Family 7: Mother and Grandmother have OI	1	Yes	No	She was born at term via a Cesarean section due to fetal distress, following a pregnancy complicated by gestational cholestasis. Her neuromotor development is within normal range.
8	04/22	Male	8	Family 4: Mother has Breast Cancer	1	Yes	Yes	At birth, the proband had a birth weight of 3,010 g and a birth length of 50 cm. Neonatal assessments revealed femoral bowing and facial dysmorphisms. Standard karyotyping and CGH array analysis (250 kb) yielded normal results. However, the proband was discharged with a suspected diagnosis of OI. At the age of 5, a femur fracture was reported. Consolidated outcomes of a previous distal tibial metaphyseal fracture with a slightly curved appearance of the mid-distal diaphyseal segment of the fibula, which also appears to be thinned, likely due to residual plastic deformation, are observed in the proband. The proband also presents with diffuse osteopenia and more pronounced cortical thinning, primarily in the periarticular region. The Z-score measured at the lumbar spine level is approximately −4.1, indicating a compatible condition of osteoporosis.
9	05/22	Male	15	Family 5: Father has OI (paz 06/22)	1	Yes	No	No supplementary clinical details
10	06/22	Male	57	Family 5: Son has OI (paz 05/22)	1	Yes	No	No supplementary clinical details

**Figure 1:**
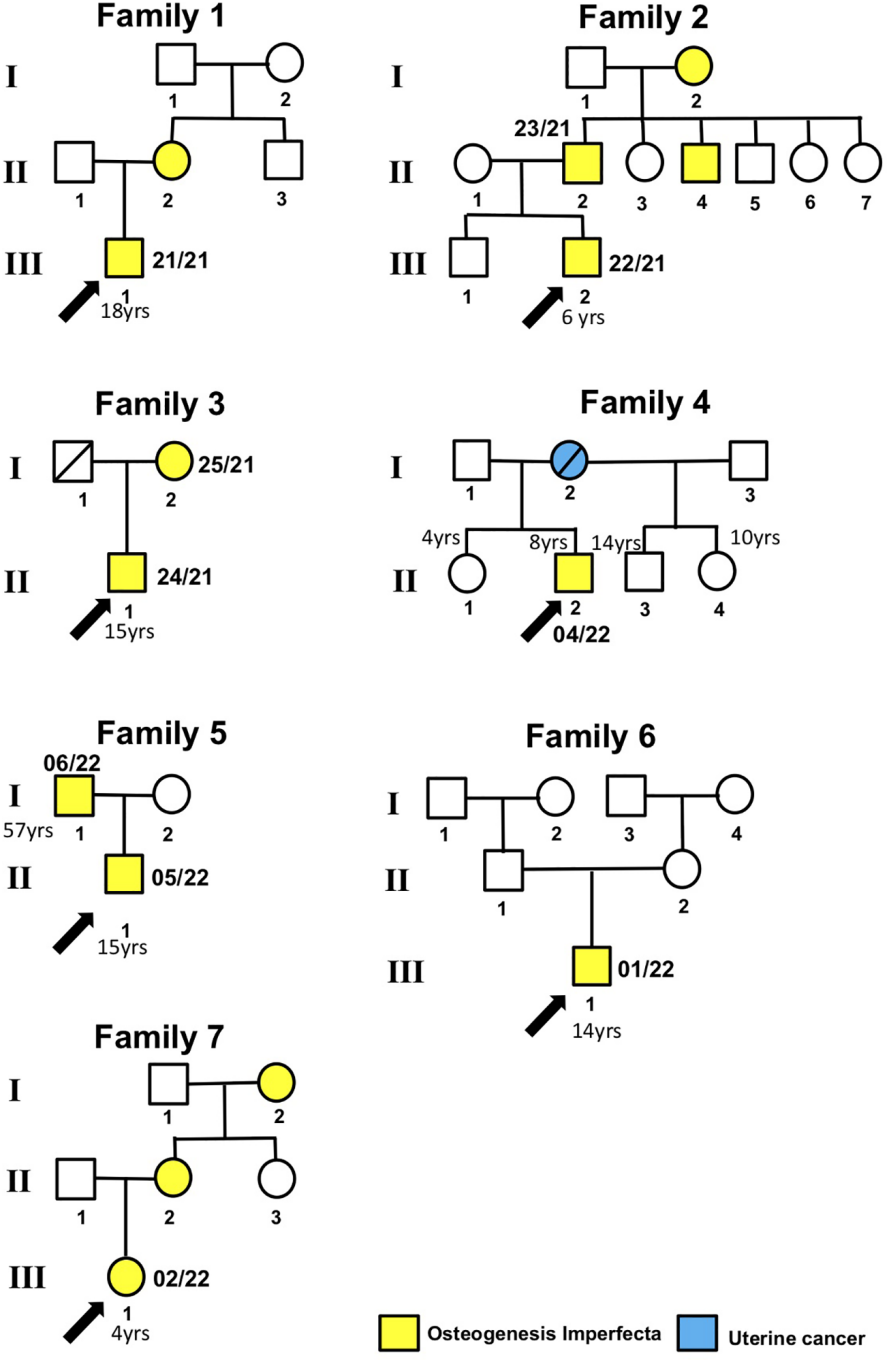
Patient pedigree trees. Probands and family members whose samples were available for this study are indicated by arrows and numbers (internal code), respectively.

This study included 10 affected individuals from seven Italian families clinically characterized as type I OI. The pedigrees of the families are shown in **Figure 1**.

### Clinical features: family 1

The proband 21/21 (III.1) was an 18-year-old Italian man, the only son of non-consanguineous parents, a normal pregnancy lasting 9 months, and a Cesarean section due to severe joint limitation, weighing 3.16 kg and artificial feeding. The mother has OI, diagnosed at the age of 6. The patient has suffered from numerous fractures with functional outcomes. X-ray radiography of the upper and lower extremities was examined for patient 22/21 (**Supplementary Image 1**).

### Clinical features: family 2

The proband 22-21 (III.2) was a 6-year-old Italian boy. The historical medical background is as follows: the proband was the second-born child, with a father (patient 23/21, II.2) who had OI and a history of limb fractures. The proband’s uncle (II.4) and grandmother (I.2) were also affected by OI, while the proband’s mother and older brother were healthy.

### Clinical features: family 3

Proband 24/21 (II.1) was a 15-year-old boy, the only son of non-consanguineous parents (**Figure 1**). The mother (patient 25/21: I.2) was affected by OI. His biological father died at 29 years of age due to cardiac ischemia.

### Clinical features: family 4

The proband 04-22 (II.2) was a 8-year-old male subject. He was born as the third child to a mother aged 28, who unfortunately passed away due to uterine cancer. The family history includes a younger sister (II.1) aged 4, exhibiting apparent good health; an older half-sister (II.4) aged 10, also in apparent good health; and an older half-brother aged 14 (II.3), conceived with a previous husband, and similarly in apparent good health. The father, aged 38, showed no notable health concerns.

### Clinical features: family 5

The proband 05/22 (II.1) was a 15-year-old Italian boy. The patient was admitted due to a right olecranon fracture associated with OI. Additionally, the patient’s 57-year-old father is also affected by OI.

### Clinical features: family 6

Proband 01/22 (II.1) was a 14-year-old girl who was the firstborn in her family. She was diagnosed with OI and exhibited blue sclerae since the age of 2. The proband experienced her first fracture in the left tibia at 1 year of age, subsequently followed by fractures in the right ulna and tibia at 2 years, and the right metatarsal at 3 years old. There were no family members affected with OI.

### Clinical features: family 7

Subject 02/22 (III.1) was a 4-year-old Italian girl with OI, and her mother, aged 27 (II.2), and her grandmother (I.2) also exhibited the same condition. Multiple fractures were reported during her infancy. At the age of 2, she was admitted to the Orthopedics Department at the hospital due to an unprovoked fracture in the right tibia and fibula. Additionally, during the same month, a metaphyseal fracture in the right femur was treated by immobilizing it with a cast. X-ray radiography of both the upper and lower extremities was conducted for patient 02/22 (**Supplementary Image 2**).

### Genetic testing and variants distribution

Ten patients from seven different OI families were analyzed by a targeted NGS panel for the common OI-related genes including *COL1A1, COL1A2, FKBP10, IFITM5, PPIB, SERPINF1, WNT1, BMP1, CRTAP, P3H1*, and *SERPINH1*. Among the 10 OI-positive patients, 7/10 PVs were found in the *COL1A1* whereas 3/10 were in the *COL1A2* gene. The PVs identified in *COL1A1* were c.1767 + 1G>A, c.658C>T, c.2890_2893del, c.517G>T, and c.3887del, whereas in *COL1A2*, they were c.1009G>A and c.596G>T. Therefore, the majority of PVs were found in *COL1A1* (*n* = 5), followed by *COL1A2* (*n* = 2) (**Table 2**). No likely pathogenic (LPVs) or PVs were detected in *FKBP10, IFITM5, PPIB, SERPINF1, WNT1, BMP1, CRTAP, P3H1*, and *SERPINH1* genes.

**Table 2:** List of pathogenic variants identified in this study.

Patients	Gene ID	dbSNP	Variant (HGVS) GRCh37	Type of variant	Clinvar classification/accession number	MAF GnomAD%	Reference
21/21	***COL1A1***	NR	Chr17:g.48271303C>T NM_000088.4:c.1767 + 1G>A	Splice site	Pathogenic	NR	(18–23)
22/21 23/21	***COL1A1***	rs72667036	Chr17: g.48275131G>A NM_000088.4:c.658C>T (p.Arg220ter)	Missense	Pathogenic	0.00000412	(24, 25)
24/21 25/21	***COL1A1***	NR	Chr17: g.48266578_48266581del NM_000088.4: c.2890_2893del (p.Pro964ValfsTer143)	Frameshift	NR	NR	NR
01/22	***COL1A1***	rs193922157	Chr17:g.48275820C>A NM_000088.4: c.517G>T (p.Gly173Ter)	Nonsense	Pathogenic/likely pathogenic VCV000035924.4	NR	(18–23)
02/22	***COL1A1***	NR	Chr17:g.48263797del NM_000088.4: c.3887del (p.Gly1296ValfsTer35)	Frameshift	NR	NR	NR
04/22	***COL1A2***	Rs67865220	Chr7:g.94039107G>A NM_000089.4:c.1009G>A (p.Gly337Ser)	Missense	Pathogenic RCV002279258 RCV001553203 RCV002230964 RCV001250519 RCV000993573 RCV000987924 RCV000490720	NR	(26–31)
05/22 06/22	***COL1A2***	NR	Chr7:g.94037160G>T NM_000089.4:c.596G>T (p.Gly199Val)	Missense	NR	NR	NR

Minor allele frequency (MAF), Clinical variation database (www.clinvar.com); Human Genome Variation Society (www.lovd.nl); non-reported (NR).

Proband 21/21 (Family 1, III.1), an 18-year-old male patient affected by OI, was found to have the heterozygous PVs c.1767 + 1G>A in the *COL1A1* gene (**Figure 1**; **Table 2**). The proband’s mother (II.2) also affected by OI did not receive any genetic testing. The splice-site *COL1A1* variant near exon 25 of 51 (c.1767 + 1G>A) detected in patient number 21/21 has been reported to ClinVar as pathogenic (VCV001380204.2). This splice-site variant disrupts the triple-helix domain of *COL1A1* and has been associated with OI in several studies (18–21, 23).

The proband 22/21 (Family 2, III.2) is a 6-year-old boy affected by OI, with his father 23/21 (II.2), uncle (II.4), and grandmother (I.2) also affected by OI. Patients 22/21 and 23/21 were found to have the heterozygous PVs c.658C>T (p.Arg220ter) in the *COL1A1* gene (**Figure 1**). The *COL1A1* nonsense variant c.658C>T (p.Arg220ter) in exon 9 of 51 found in patients 22/21 and 23/21 was classified as pathogenic in Clinvar (VCV000425637.13) and described to be associated with OI in several studies (24, 32–35).

Patient 24/21 (Family 3, II.1) and his mother 25/21 (I.2) both affected by OI were found to have the heterozygous variant c.2890_2893del (p.Pro964Valfs*143) in the *COL1A1* gene. To our knowledge, this *COL1A1* frameshift deletion in exon 40 of 51 has not been reported previously as a PV or as a benign variant, and it is predicted to cause loss of normal protein function through protein truncation caused by a frameshift mutation.

Patient 01/22 (Family 6, III.1) affected by OI was found to have the pathogenic heterozygous variant c.517G>T (p.Gly173Ter) in the *COL1A1* gene. This sequence change in exon 6 of 51, which creates a premature translational stop signal (p.Gly173*) in the *COL1A1* gene, has been observed in individuals with OI (36) and ClinVar contains an entry for this variant (Variation ID: 35924).

Patient 02/22 (Family 7, III.1) affected by OI who had clinical manifestations typical of OI, was a heterozygous carrier of the c.3887del (p.Gly1296ValfsTer35) in the *COL1A1* gene. The proband’s mother (II.2) and grandmother (I.2) also affected by OI did not receive any genetic testing. Currently, this *COL1A1* frameshift variant in exon 49 of 51 has not been previously described in publications and has not been registered in any databases.

The proband 04/22 (Family 4, II.2), an 8-year-old boy who had clinical manifestations typical of OI, was a heterozygous carrier of the c.1009G>A (p.Gly337Ser) in the *COL1A2* gene (**Figure 1**). The proband’s mother (Family 4, I.2), who died due to ovarian cancer, and two more proband’s sister (Family 5, II.1 and II.4) and one proband’s half-brother (Family 4, II.4), who were 10, 4, and 14 years old, respectively, and who were referred to be healthy, did not receive any genetic testing. This *COL1A2* missense variant in exon 19 of 52 has been observed in individuals with OI types I, III, and IV (24, 29, 30, 33, 37–43).

The proband 05/22 (Family 5, II.1) is an 8-year-old boy affected by OI, and his father 06/22 (Family 5, I.1) also had clinical manifestations typical of OI (**Figure 1**; **Table 2**). After NGS genetic analysis, they were both found to be heterozygous carriers of the c.596G>T (p.Gly199Val) missense variant in exon 13 of the *COL1A2* gene (**Figure 1**). Notably, the PV was present only in affected patients. Currently, this *COL1A1* missense variant has not been previously registered in any databases and has not been described in publications.

### Interpretation of the novel PVs identified in OI patients

To our knowledge, the *COL1A1* frameshift variant c.2890_2893del (p.Pro964ValfsTer143) identified in patient 24/21 and his mother 25/21 has not been reported previously as a PV. The p.Pro964Valfs*143 variant is novel in gnomAD and 1kG database, and it is predicted to cause loss of normal protein function through protein truncation caused by a frameshift mutation. The *COL1A1* frameshift variant identified here was classified as likely pathogenic based on the American College of Medical Genetics (ACMG) criteria (PVS1 and PM2) (44) (**Table 3**).

**Table 3:** Interpretation of novel identified likely pathogenic variants using ACMG rules.

n.	Patient ID	Gene	Variant	ACMG criteria	Interpretation
**1**	**24/21** **25/21**	***COL1A1***	c.2890_2893del (p.Pro964ValfsTer143)	**PVS1, PM2**	**Likely pathogenic**
**2**	**02/22**	***COL1A1***	c.3887del (p.Gly1296ValfsTer35)	**PVS1, PM2**	**Likely pathogenic**
**3**	**05/22** **06/22**	***COL1A2***	c.596G>T (p.Gly199Val)	**PM1, PM2, PP2, PP3, PM5**	**Likely pathogenic**

To our knowledge, the frameshift deletion c.3887delG (p.Gly1296Valfs*35) in *COL1A1* found in patient 02/22 has not been reported previously as a PV or as a benign variant as well. The p.Gly1296Valfs*35 variant is novel in gnomAD and 1kG database, and it is predicted to cause loss of normal protein function through protein truncation caused by a frameshift mutation. This variant is a frameshift variant that occurs in an exon of *COL1A1* upstream where nonsense-mediated decay is predicted to occur. The *COL1A1* frameshift variant identified here was classified as likely pathogenic based on the ACMG criteria (PVS1, and PM2) (44) (**Table 3**).

**Figure 2:**
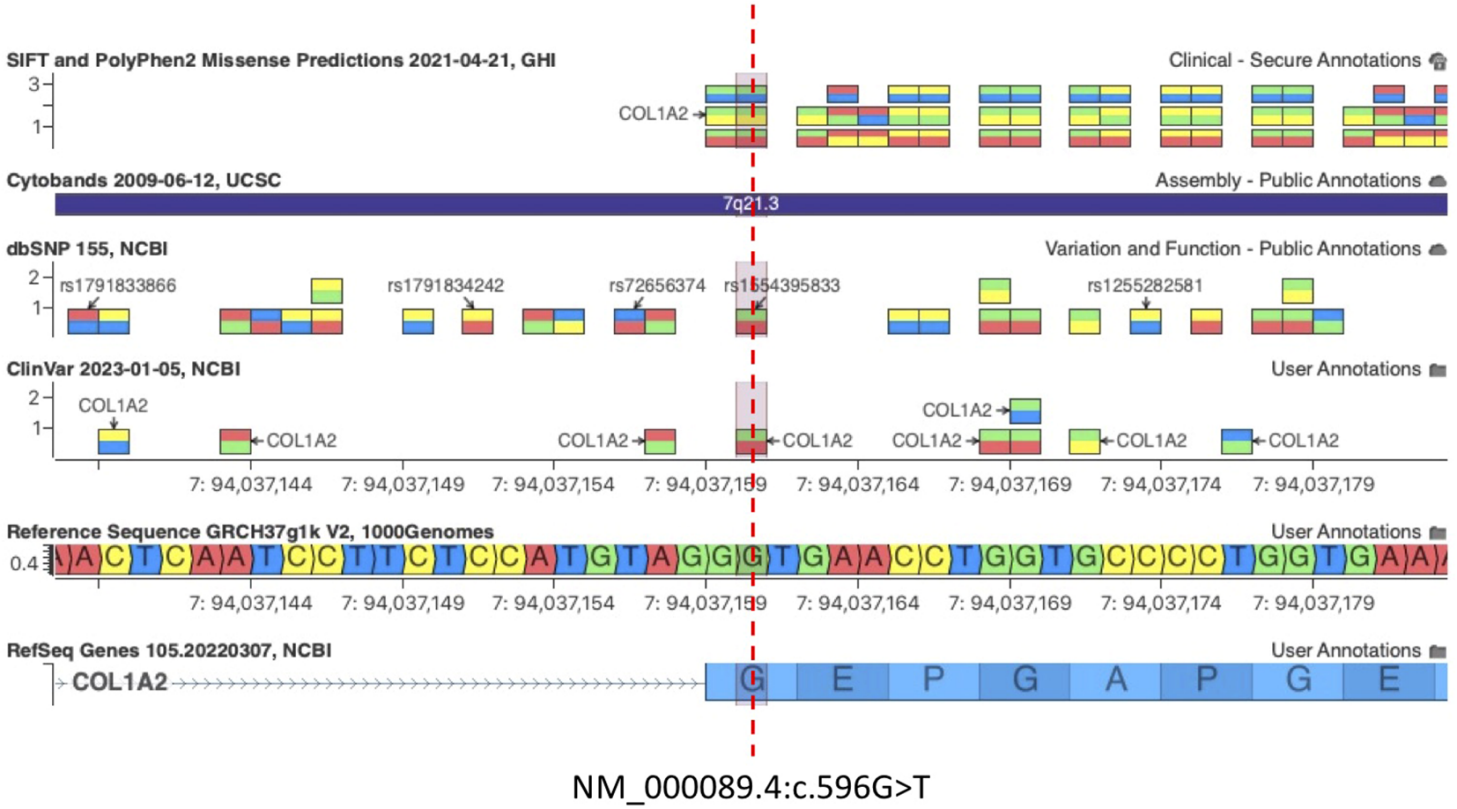
c.596G>T *COL1A2* variant is predicted to be damaging by both SIFT and PolyPhen2. The pathogenic variant c.596G>A (Gly199Asp) at the same amino acid residue of COL1A2 was associated with OI, confirming that Glycine 199 (G199) is an essential residue for COL1A2 function [Clinvar VCV000496615.30, dbSNP 1554395833].

The missense variant c.596G>T(p.G199V) in the *COL1A2* gene detected in patients 05/22 and his father 06/22 was not observed in the large population cohorts of gnomAD or 1kG database (Genome Aggregation Database and Genomes Consortium) (**Figure 2**). In addition, the computational tools used to evaluate the possible pathogenicity of this variant showed that the c.596G>T *COL1A2* variant is predicted to be damaging by both SIFT and PolyPhen2. The *COL1A2* missense variant identified here, not previously described in any database including HGMD, LOVD, and ClinVar, was classified as likely pathogenic based on the ACMG criteria (PM1, PM2, PP2, PP3, and PM5) (44) (**Table 3**).

Importantly, the c.596G>T (p.Gly199Val) variant on exon 13 of *COL1A2* is not predicted to disrupt the existing acceptor splice site 2 bp upstream by any splice site algorithm such as GeneSplicer, MaxEntScan, NNSplice, PWM, and VarSeak (**Supplementary Image 3**), whereas HSF PRO showed a significant alteration of ESE/ESS motifs ratio, suggesting that this mutation could have a potential impact on splicing (**Supplementary Table 1**). A PV c.596G>A (Gly199Asp) at the same amino acid residue of *COL1A2* was associated with OI, confirming that Glycine 199 (G199) is an essential residue for COL1A2 function (**Figure 2**, Clinvar VCV000496615.30, dbSNP 1554395833). Importantly, the glycine residue at codon 199 of *COL1A2* is conserved in all mammalian species (**Supplementary Image 4**). Snapshots of the Integrative Genomics Viewer (IGV) showing all the germline variants identified in this study can be found in the Supplementary Material (**Supplementary Image 5**).

## Discussion

Despite extensive research, our understanding of the complete genetic landscape of OI remains incomplete. To comprehensively analyze the relationship between the genotype and phenotype of OI, it is of significant importance to confirm OI patients and discover new PVs through genetic tests (45). Therefore, understanding the genetic basis of OI is crucial for accurate diagnosis, genetic counselling, and potential therapeutic interventions (10). Firstly, it allows for a more precise classification of OI subtypes based on specific genetic etiology, which can aid in predicting disease severity and natural history. Secondly, it enables accurate genetic counselling, informing affected individuals and their families about the recurrence risks and providing them with information regarding prenatal or preimplantation genetic testing options. Lastly, the discovery of rare PVs expands the potential targets for therapeutic interventions, as specific genetic defects can be targeted with gene-based therapies or other targeted approaches (46).

Our study aims to expand the genetic and clinical spectrum of OI by identifying rare PVs in the commonly implicated *COL1A1* and *COL1A2* as well as in other genes involved in collagen metabolism, osteoblast differentiation, and bone mineralization. Variants in *COL1A1* and *COL1A2* genes give rise to two primary classes of collagen defects: quantitative and structural defects (7). Among these, the latter, which involve alterations in collagen structural framework, tend to induce more severe phenotypic expressions due to an excessive occurrence of posttranslational modifications (47). The mechanistic underpinnings of collagen defects can be delineated into two fundamental types. Mutations leading to haploinsufficiency characterize the first category. This is typified by the synthesis of a single *COL1A1* allele, often triggered by mechanisms such as nonsense-mediated mRNA decay or the introduction of pre-termination codons through frameshift or splicing mutations. These mutations are prominently associated with the milder form of OI (48). The second category encompasses helical mutations occurring within *COL1A1* or *COL1A2*, resulting in structural alterations within type I collagen. Specifically, missense mutations located within the triple-helical domain exert a dominant negative effect, thereby disrupting the proper folding and synthesis of collagen. Notably, these helical mutations frequently involve substitutions of glycine residues and manifest a spectrum of severity, ranging from mild to profound (48, 49).

By utilizing a targeted gene panel and comprehensive clinical data, we identified two novel heterozygous frameshift PVs in *COL1A1* (c.2890_2893del and c.3887del) and one novel heterozygous missense PV in *COL1A2* (c.596G>T), whereas no PVs were found in other OI-related genes. The heterozygous PVs c.2890_2893del (p.Pro964Valfs*143) and c.3887del (p.Gly1296ValfsTer35) variants in the *COL1A1* gene, located in exons 40 and 49, respectively, both result in frameshift deletions. These variants have not been previously reported as pathogenic and were predicted to cause a loss of normal protein function through protein truncation. This category of mutations typically induces premature termination codons (PTCs) within the coding sequence of a single allele of *COL1A1*. Such mutations initiate a process known as nonsense-mediated decay (NMD), leading to the degradation of the corresponding mRNA (21, 50, 51). Mutations provoking PTCs within *COL1A1* culminate in mRNA instability, concomitantly diminishing the production of normal type I procollagen, thereby resulting in the mild type I OI phenotype (21).

To the best of our knowledge, the c.596G>T (p.Gly199Val) missense variant in exon 13 of the *COL1A2* gene has not been previously registered in any OI databases and has not been described in publications as well. Since this variant is in close proximity from the splice site, it was suspected to affect *COL1A2* splicing. Thus far, a total of 858 PVs have been cataloged within the *COL1A2* gene (https://databases.lovd.nl/shared/genes/COL1A2). Among these, approximately 11% of the variants have been documented as alterations occurring at splice sites, thereby constituting the second most prevalent category of mutation (52). Mutations at splice sites in *COL1A2* can lead to diverse outcomes, such as exon skipping, inclusion of intronic sequences, or the activation of cryptic sites within introns or exons (21). In some instances, nucleotide changes arising from mutations may sporadically activate cryptic splice sites, often positioned in proximity to authentic splice sites (52).

Although the sample size was limited, we conducted a thorough analysis of all genes present in the gene panel and identified variants only in the *COL1A1* and *COL1A2* genes. However, it is important to acknowledge that the low number of patients included in our study may have hindered the detection of variants in other genes. Since our study provides valuable insights into the variants identified in *COL1A1* and *COL1A2*, it is crucial to address the limitations imposed by the small sample size. Larger cohort studies are warranted to explore the potential involvement of additional genes in OI pathogenesis (5). Recent advances in next-generation sequencing technologies and the availability of larger patient cohorts have facilitated the identification of novel OI genes (10). By considering these advancements, forthcoming research can augment our comprehension of the intricate mechanisms that underlie OI and enhance clinical management strategies. For instance, studies have identified PVs in genes such as *IFITM5* (53) and *SERPINF1* (16), which play crucial roles in bone formation and mineralization. Therefore, it is essential to investigate these genes and others in future research to uncover additional genetic determinants.

Our study reinforces the significance of mutations in *COL1A1* and *COL1A2* in the pathogenesis of OI. Collagen type I, encoded by these genes, is the major structural component of bone, and alterations in its synthesis or assembly can lead to bone fragility and increased fracture risk (9). Multiple studies have reported the involvement of *COL1A1* and *COL1A2* mutations in OI (46, 52).

In conclusion, we have provided insights into novel PVs primarily identified in *COL1A1* and *COL1A2* genes of patients affected by OI type I. Our study validates that all of these variants are causative for OI type I, and the data of this study enrich the mutation database of OI. This study represents an important step forward in unraveling the genetic and clinical complexity of OI and highlights the significance of investigating rare PVs in OI-associated genes.

## Data availability statement

The original contributions presented in the study are publicly available. This data can be found here: BioProject database, accession number PRJNA1021801 (http://www.ncbi.nlm.nih.gov/bioproject/1021801).

## Ethics statement

Ethical approval was not required for the study involving human samples in accordance with the local legislation and institutional requirements. Written informed consent for participation in this study was provided by the participants’ legal guardians/next of kin. Written informed consent was obtained from the minor(s)’ legal guardian/next of kin for the publication of any potentially identifiable images or data included in this article.

## Author contributions

FP: Conceptualization, Data curation, Formal Analysis, Investigation, Methodology, Resources, Software, Supervision, Validation, Visualization, Writing – original draft, Writing – review & editing. RF: Data curation, Resources, Supervision, Writing – review & editing. BM: Data curation, Resources, Supervision, Validation, Writing – review & editing. DD: Conceptualization, Data curation, Resources, Supervision, Writing – review & editing. MT: Conceptualization, Resources, Supervision, Writing – review & editing.
