# Supplementary figures and images for: Expanding the genetic and clinical spectrum of osteogenesis imperfecta: identification of novel rare pathogenic variants in type I collagen-encoding genes
Source: Front Endocrinol (Lausanne). 2023 Oct 20;14:1254695. doi: 10.3389/fendo.2023.1254695 (PMC10623311; doi:10.3389/fendo.2023.1254695)

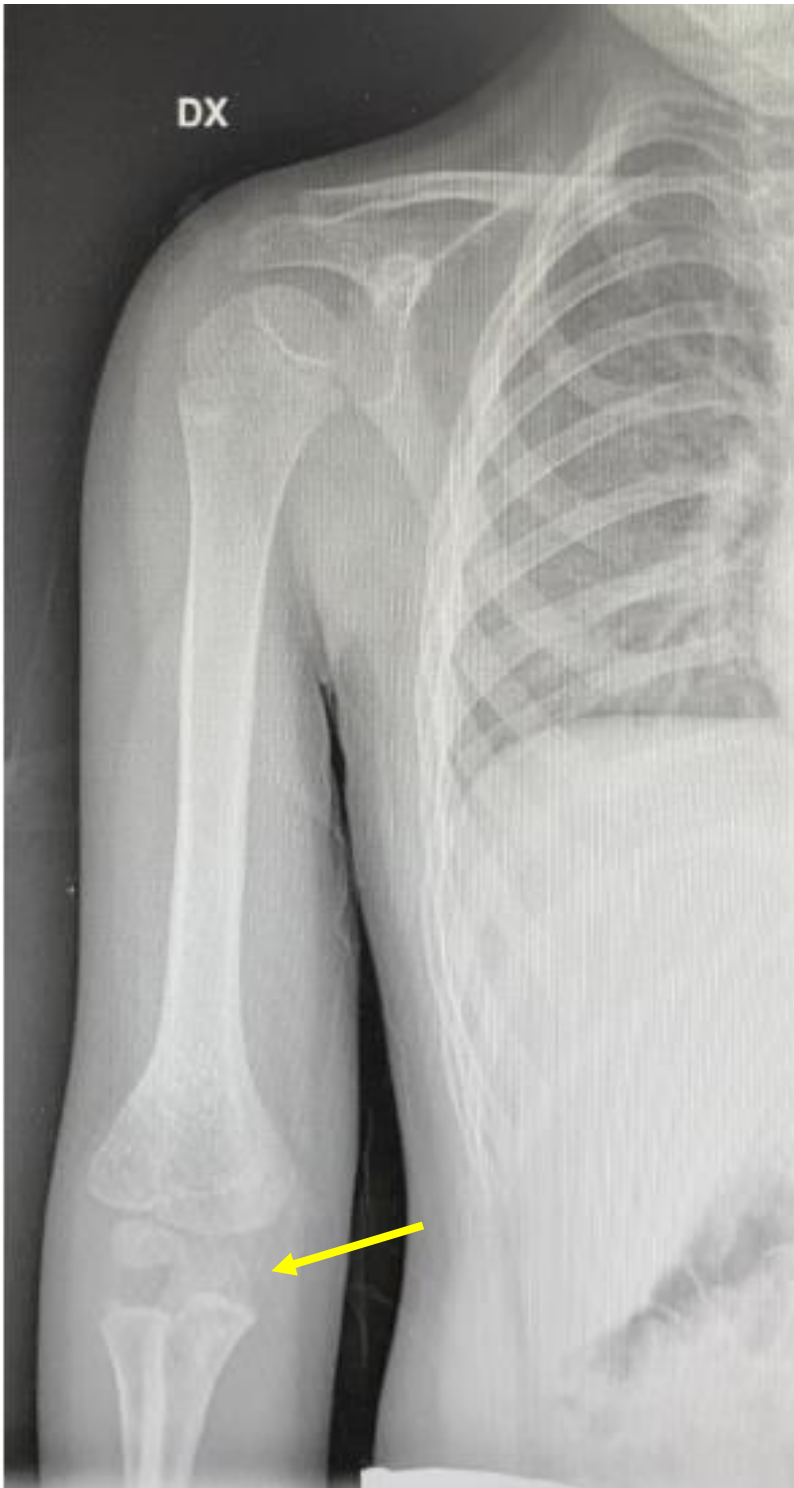

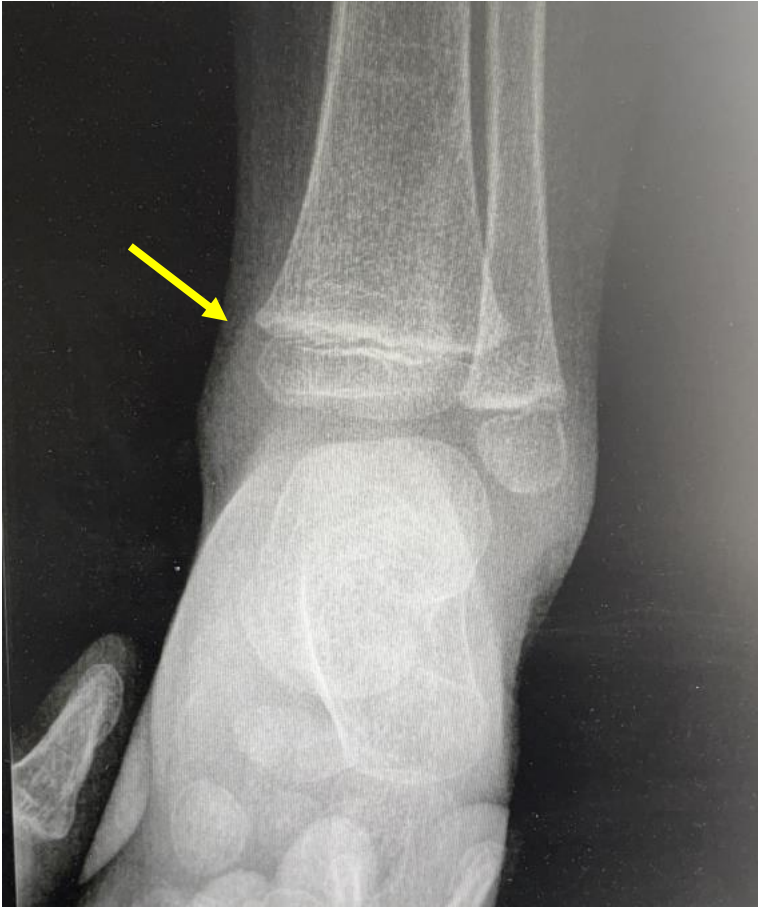

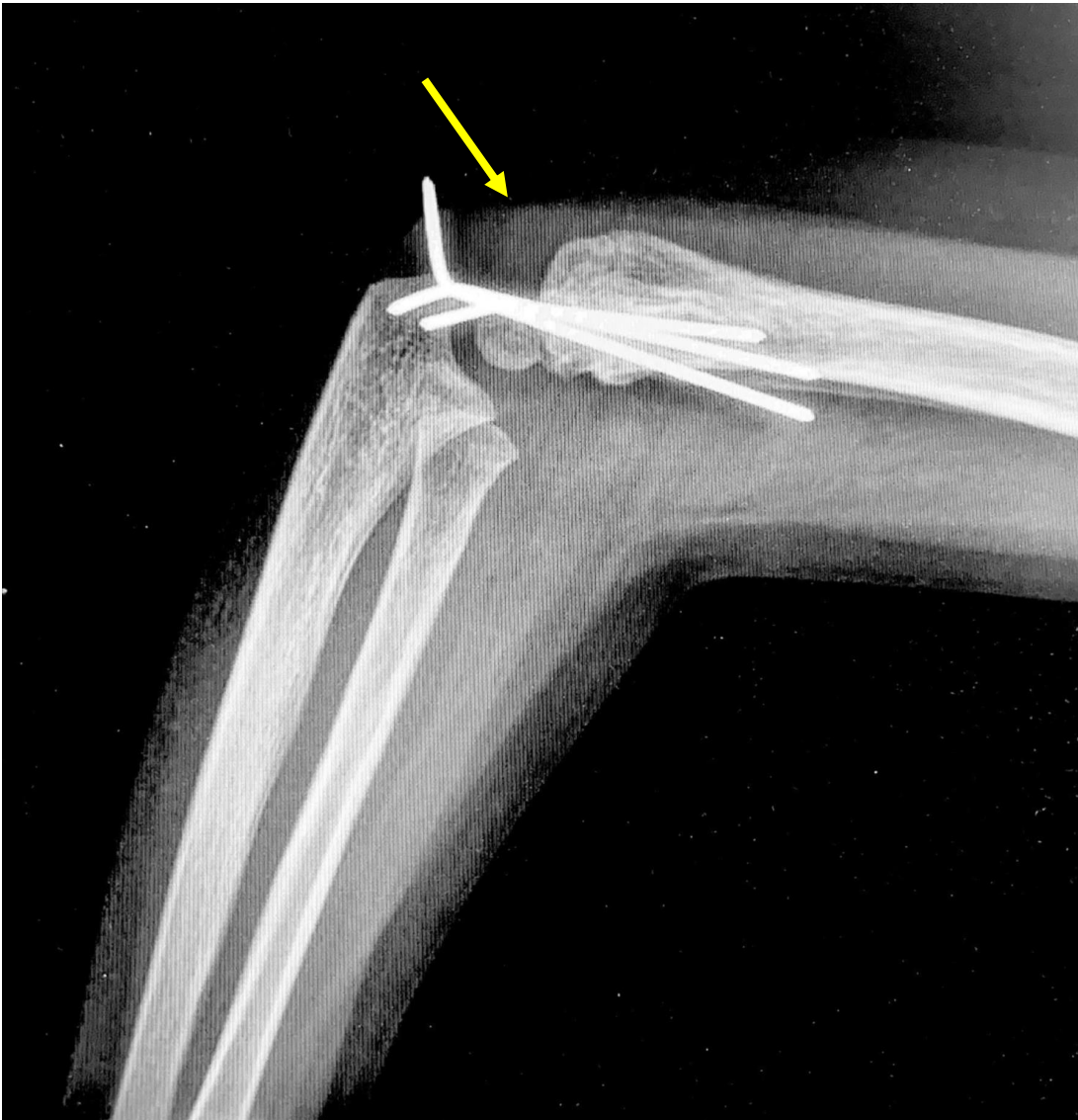

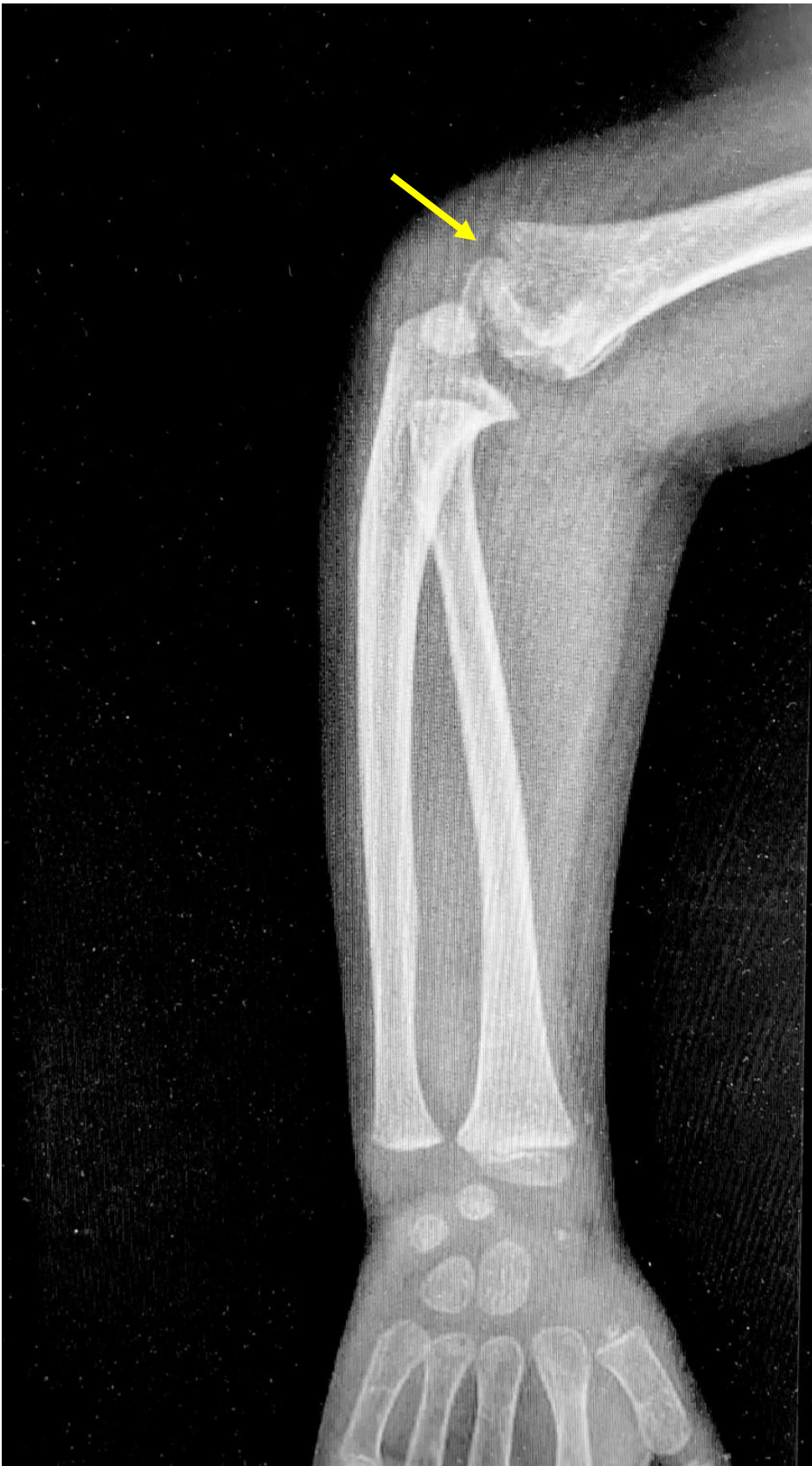

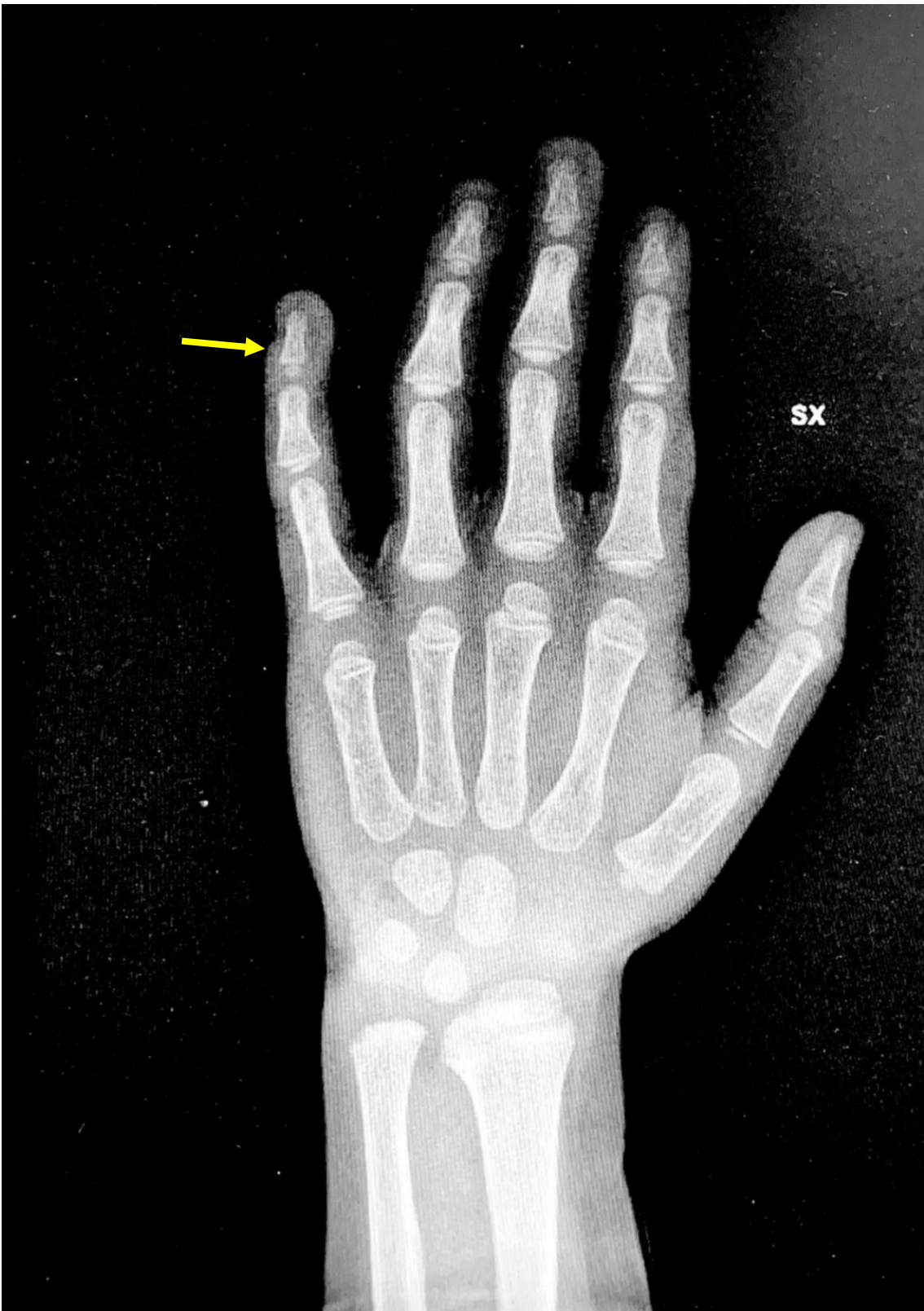

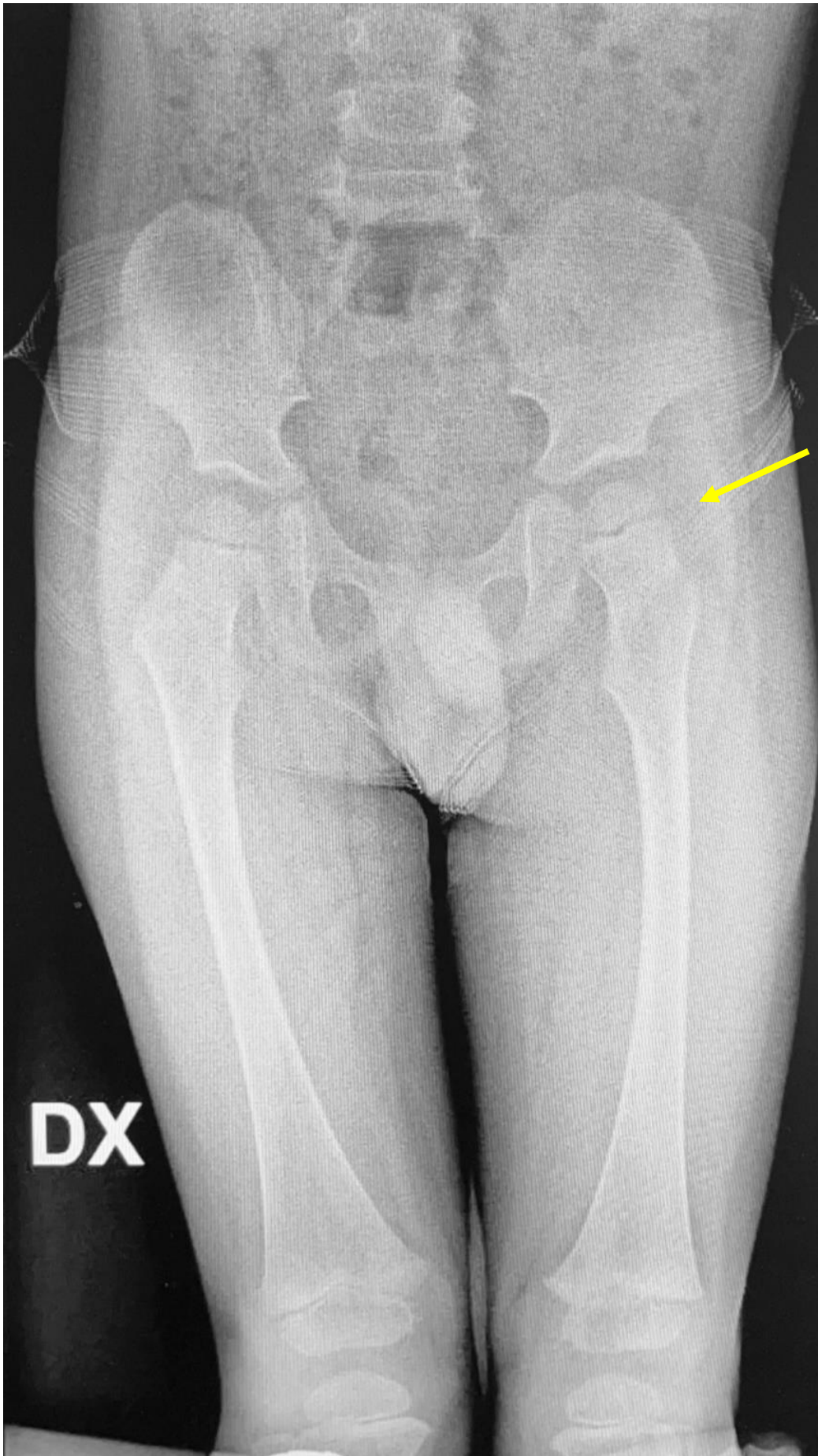

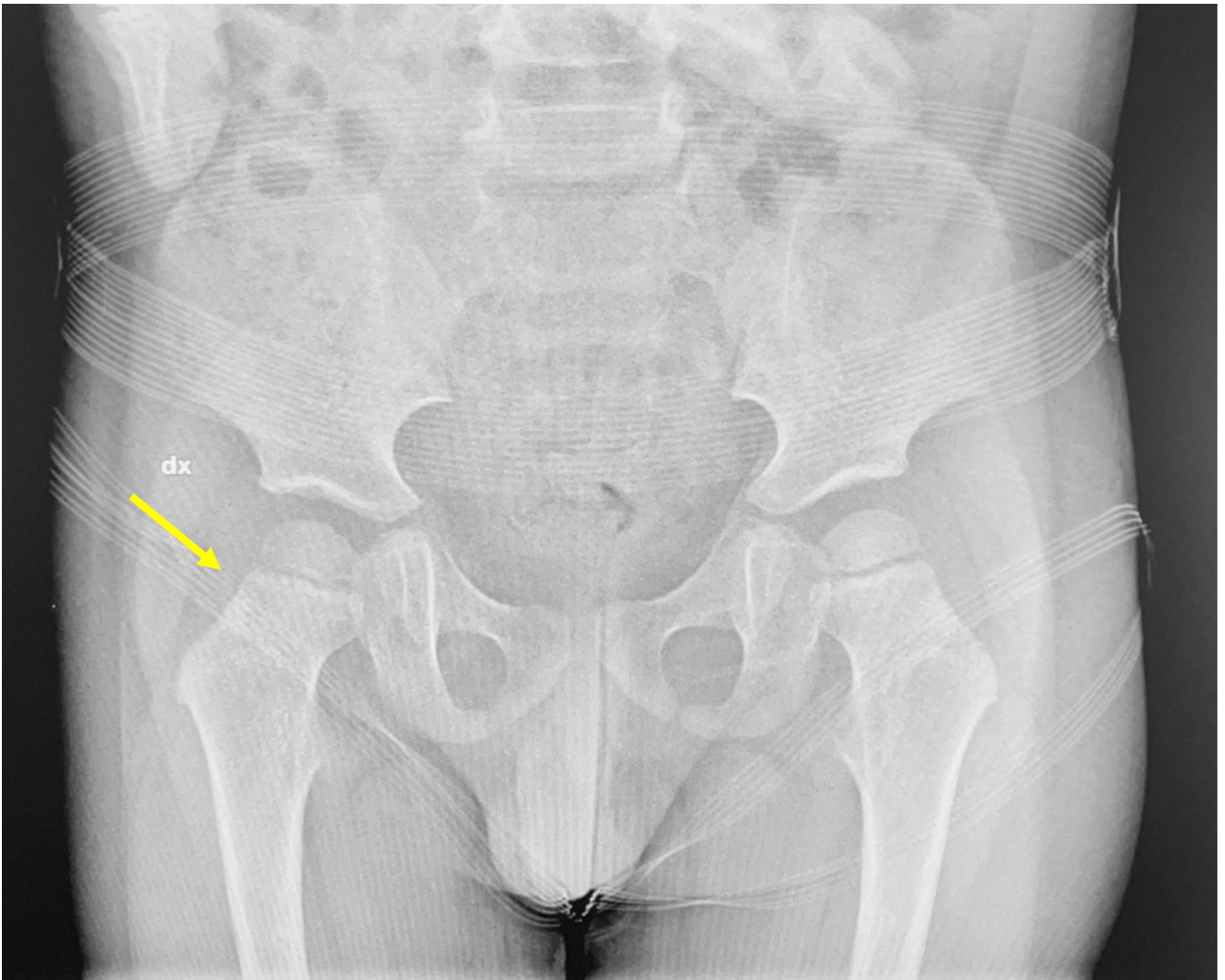

Supplement: Supplementary file 1 [file Image_1.pdf]

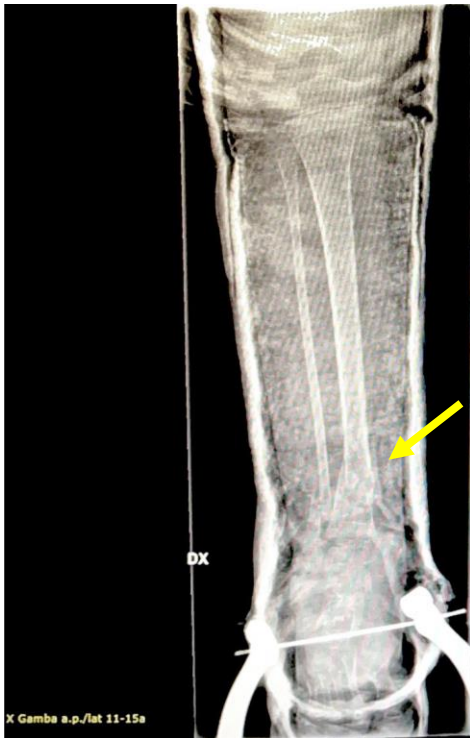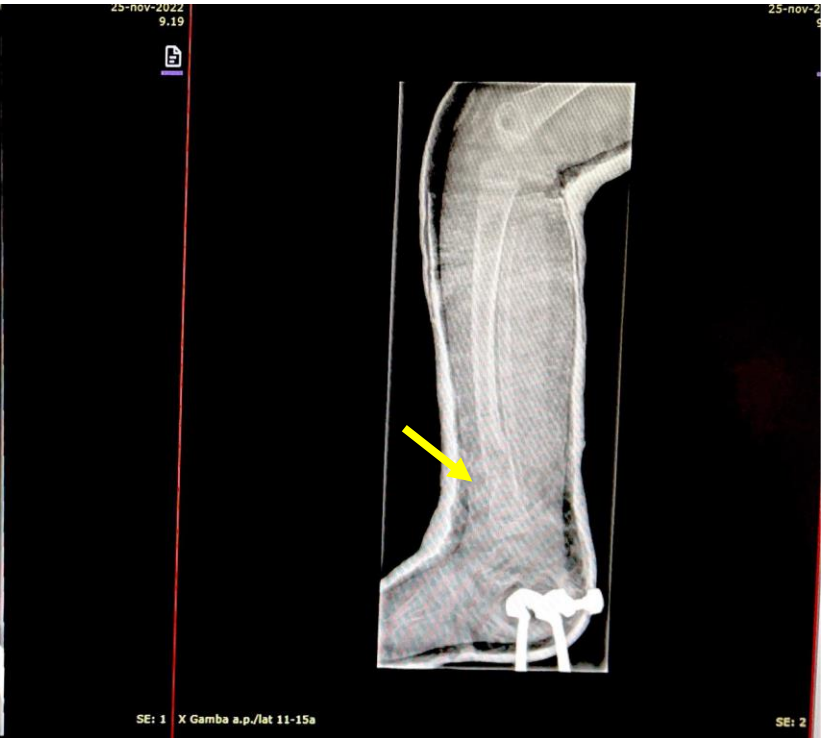

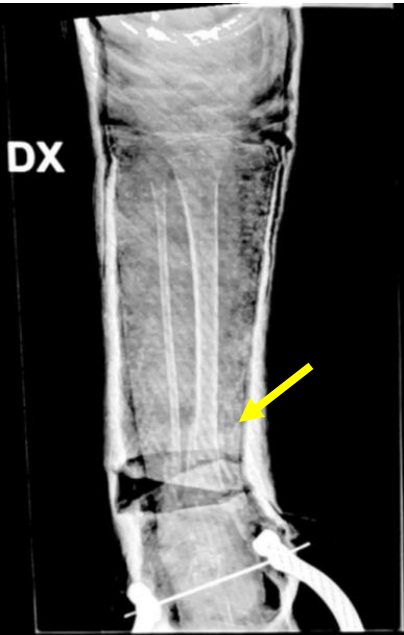

9.15  
D

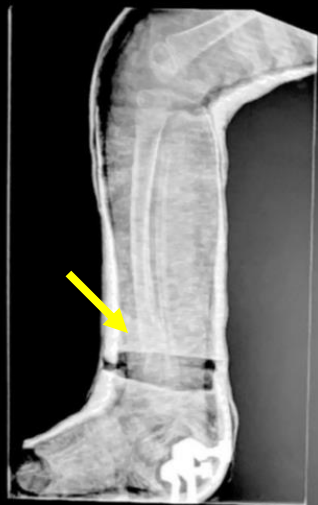



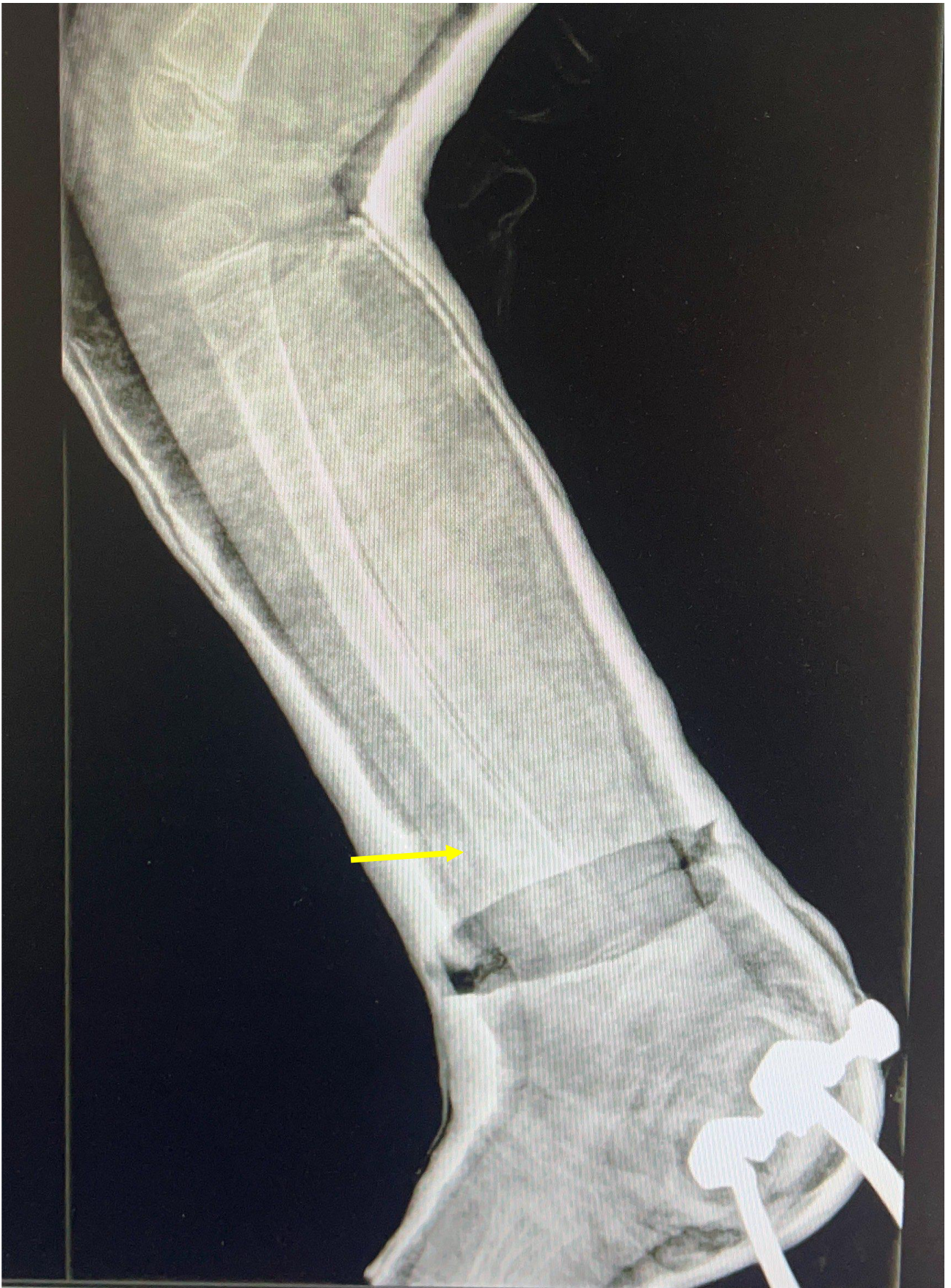

DX

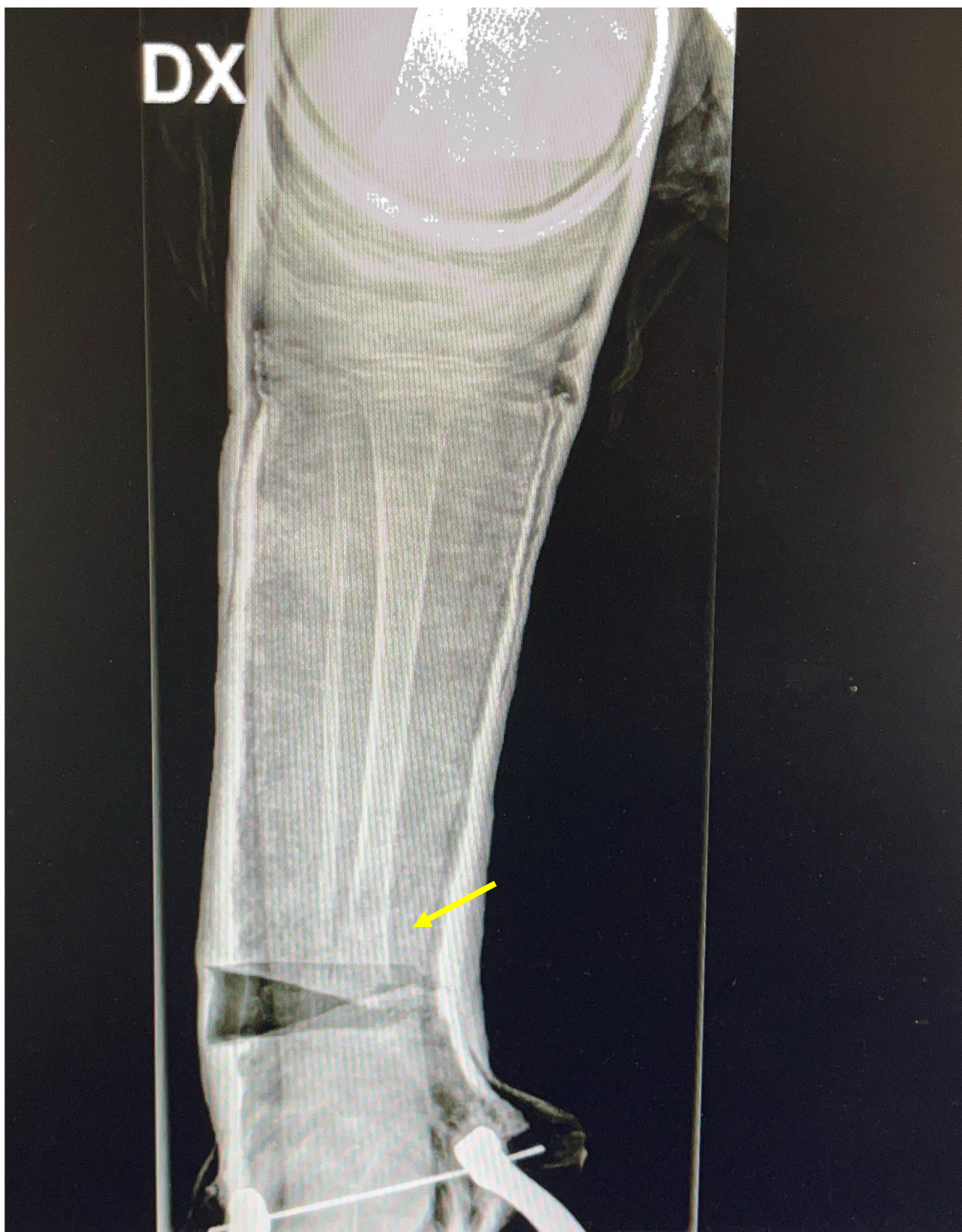

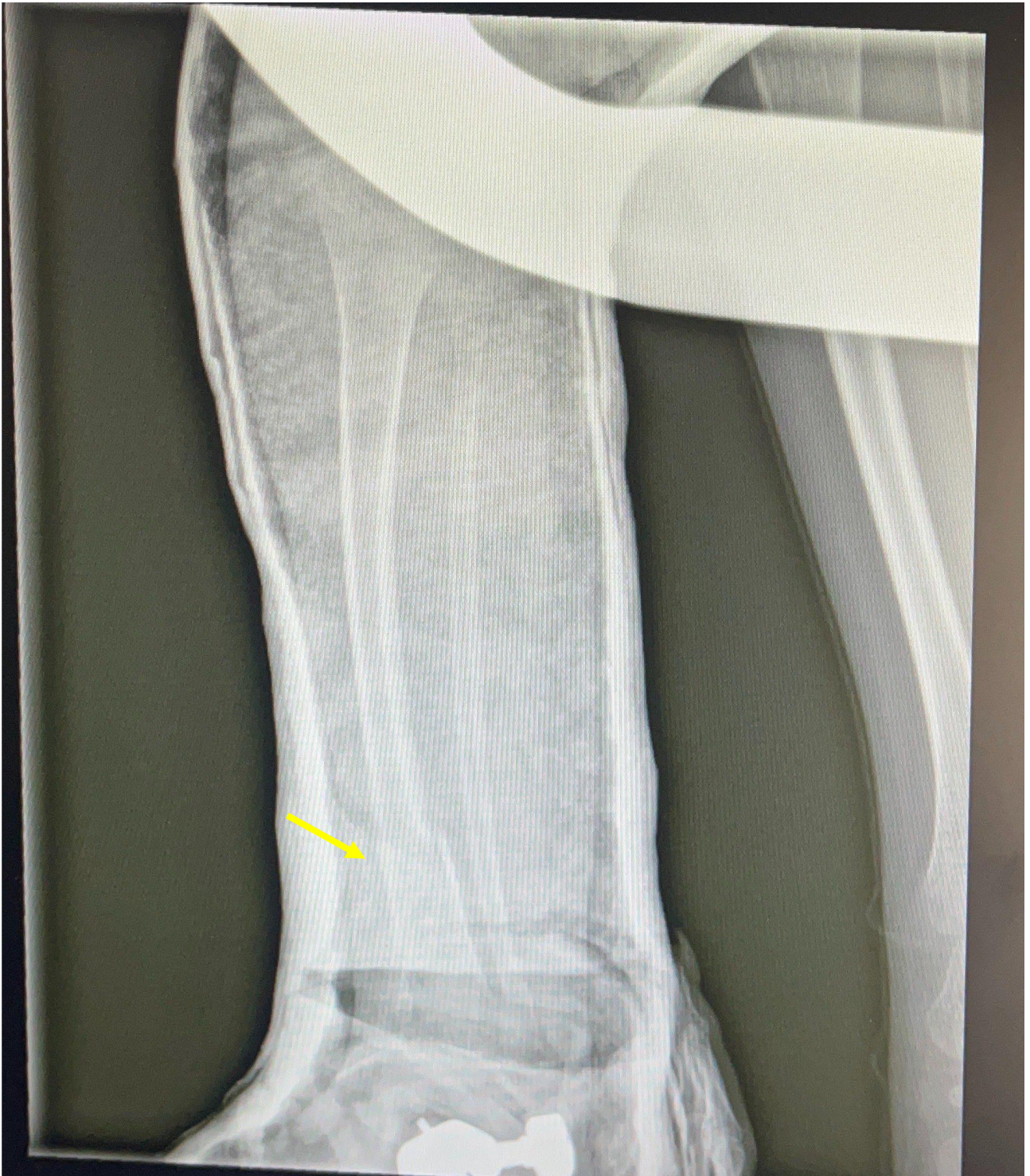

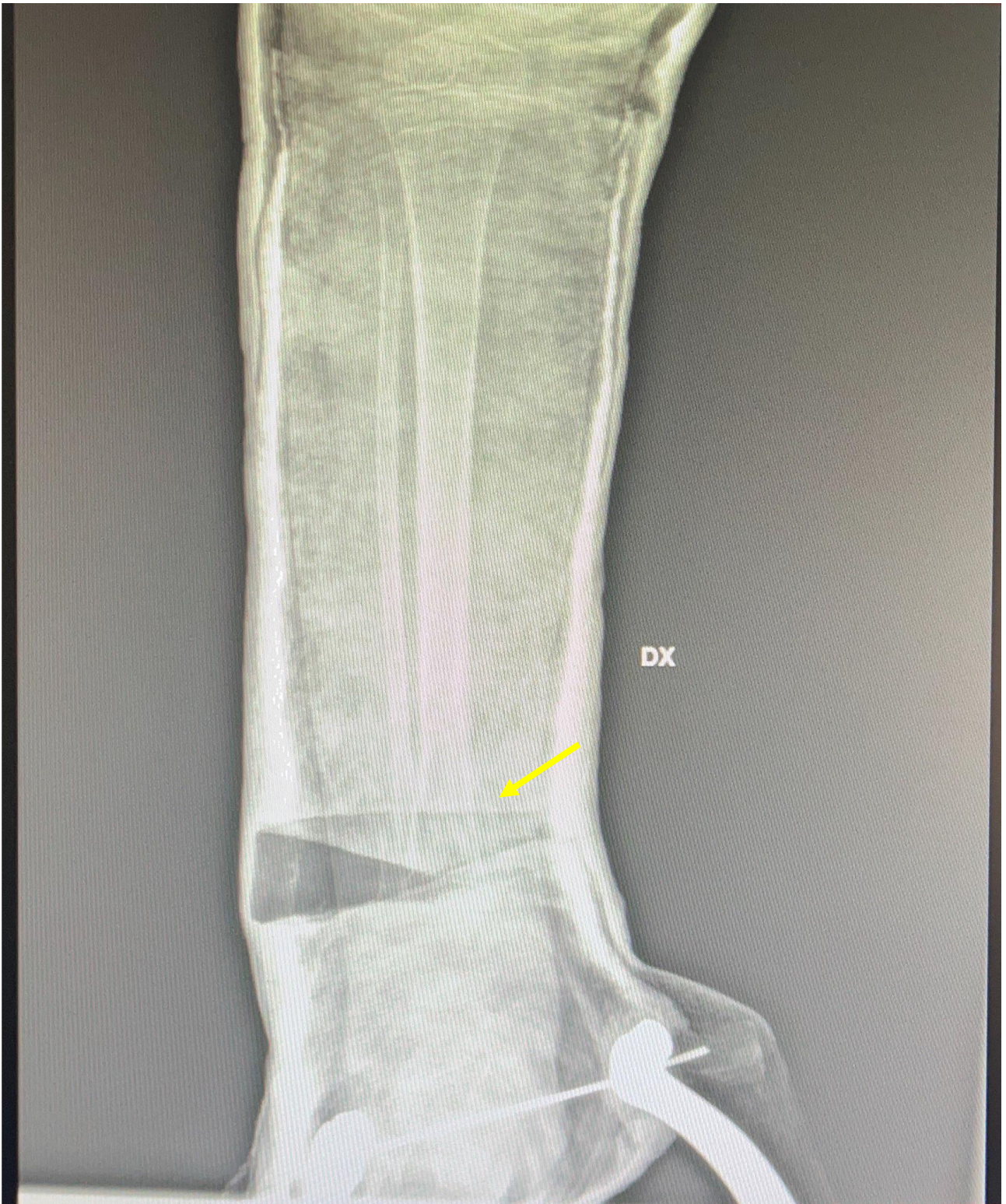

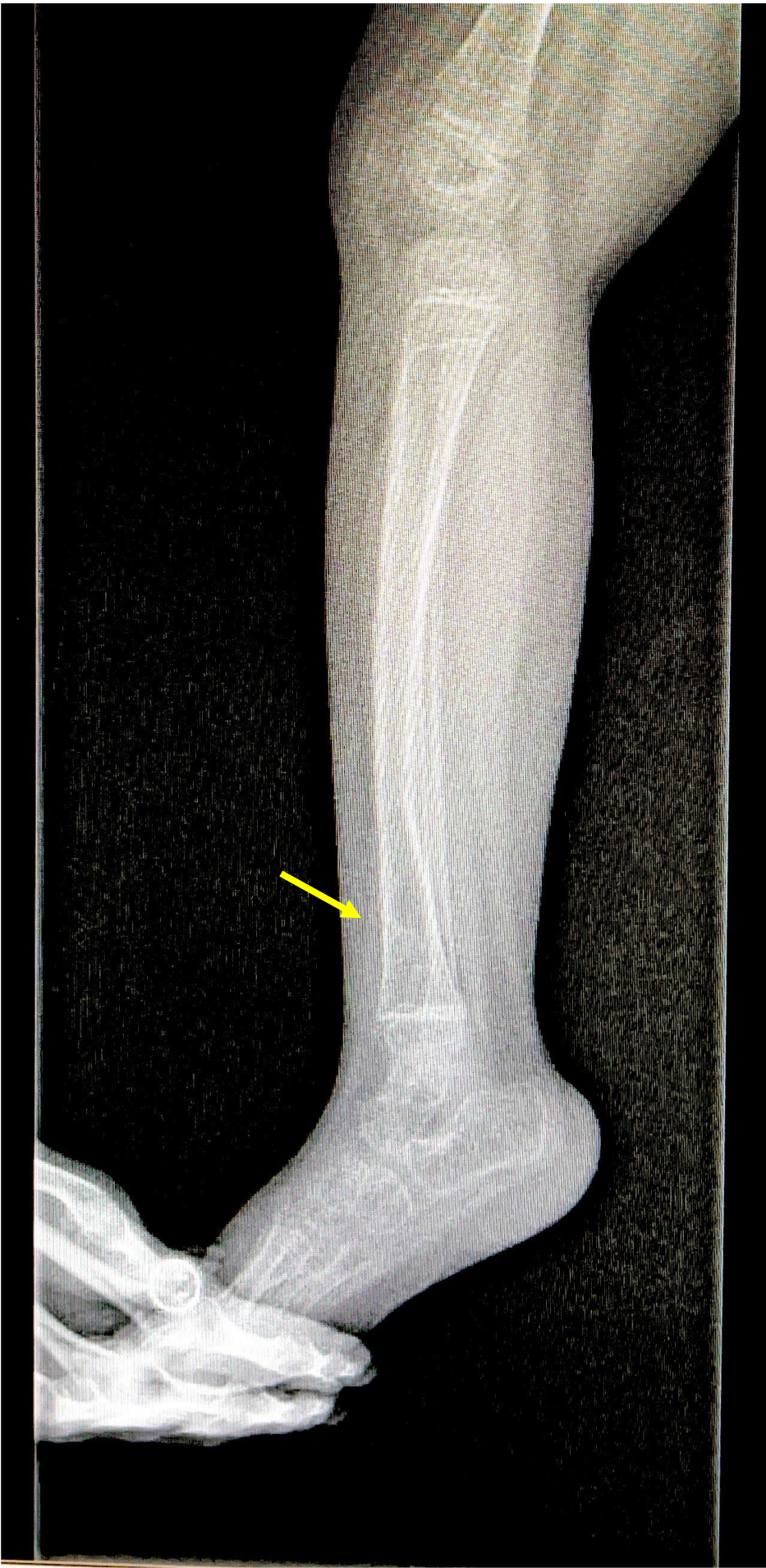

Supplement: Supplementary file 2 [file Image_2.pdf]
