# Supplementary material for: Expanding the genetic and clinical spectrum of osteogenesis imperfecta: identification of novel rare pathogenic variants in type I collagen-encoding genes
Source: Front Endocrinol (Lausanne). 2023 Oct 20;14:1254695. doi: 10.3389/fendo.2023.1254695 (PMC10623311; doi:10.3389/fendo.2023.1254695)
Supplement: Supplementary file 3 [file Image_3.pdf]

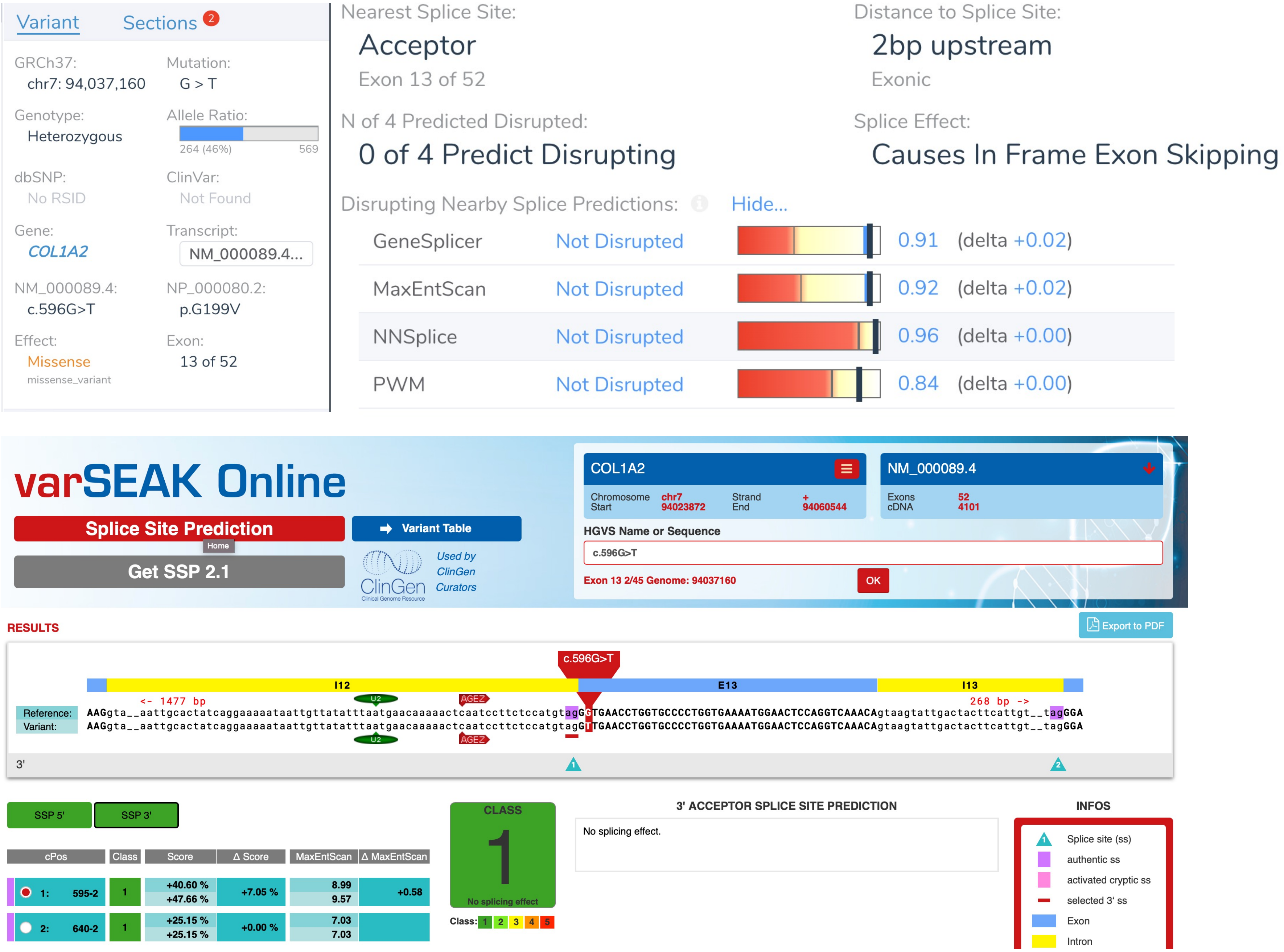

Variant
Sections 2
GRCh37:
chr7: 94,037,160
Mutation:
G > T
Genotype:
Heterozygous
Allele Ratio:
264 (46%)
569
dbSNP:
No RSID
ClinVar:
Not Found
Gene:
COL1A2
Transcript:
NM_000089.4...
NM_000089.4:
c.596G>T
NP_000080.2:
p.G199V
Effect:
Missense
missense_variant
Exon:
13 of 52
Nearest Splice Site:
Acceptor
Exon 13 of 52
Distance to Splice Site:
2bp upstream
Exonic
N of 4 Predicted Disrupted:
0 of 4 Predict Disrupting
Splice Effect:
Causes In Frame Exon Skipping
Disrupting Nearby Splice Predictions:
Hide...
GeneSplicer Not Disrupted 0.91 (delta +0.02)
MaxEntScan Not Disrupted 0.92 (delta +0.02)
NNSplice Not Disrupted 0.96 (delta +0.00)
PWM Not Disrupted 0.84 (delta +0.00)

varSEAK Online
Splice Site Prediction
Home
Get SSP 2.1
Variant Table
Used by ClinGen Curators
ClinGen
Clinical Genome Resource
COL1A2
Chromosome chr7
Start 94023872
Strand +
End 94060544
NM_000089.4
Exons 52
cDNA 4101
HGVS Name or Sequence
c.596G>T
Exon 13 2/45 Genome: 94037160
OK
RESULTS
Export to PDF
c.596G>T
I12
E13
I13
<- 1477 bp
268 bp ->
U2
AGEZ
Reference: AAGgta__aattgcactatcaggaaaaataattgttatatttaatgaacaaaaactcaatccttctccatgtagGGTGAACCTGGTGCCCCTGGTGAAAATGGAACTCCAGGTCAAACAgtaagtattgactacttcattgt__tagGGA
Variant: AAGgta__aattgcactatcaggaaaaataattgttatatttaatgaacaaaaactcaatccttctccatgtagGTTGAACCTGGTGCCCCTGGTGAAAATGGAACTCCAGGTCAAACAgtaagtattgactacttcattgt__tagGGA
3'
1
2
SSP 5'
SSP 3'
cPos | Class | Score | Δ Score | MaxEntScan | Δ MaxEntScan
1: 595-2 | 1 | +40.60 % +47.66 % | +7.05 % | 8.99 9.57 | +0.58
2: 640-2 | 1 | +25.15 % +25.15 % | +0.00 % | 7.03 7.03 |
CLASS
1
No splicing effect
Class: 1 2 3 4 5
3' ACCEPTOR SPLICE SITE PREDICTION
No splicing effect.
INFOS
Splice site (ss)
authentic ss
activated cryptic ss
selected 3' ss
Exon
Intron
