# Supplementary material for: Expanding the genetic and clinical spectrum of osteogenesis imperfecta: identification of novel rare pathogenic variants in type I collagen-encoding genes
Source: Front Endocrinol (Lausanne). 2023 Oct 20;14:1254695. doi: 10.3389/fendo.2023.1254695 (PMC10623311; doi:10.3389/fendo.2023.1254695)
Supplement: Supplementary file 4 [file Image_4.pdf]

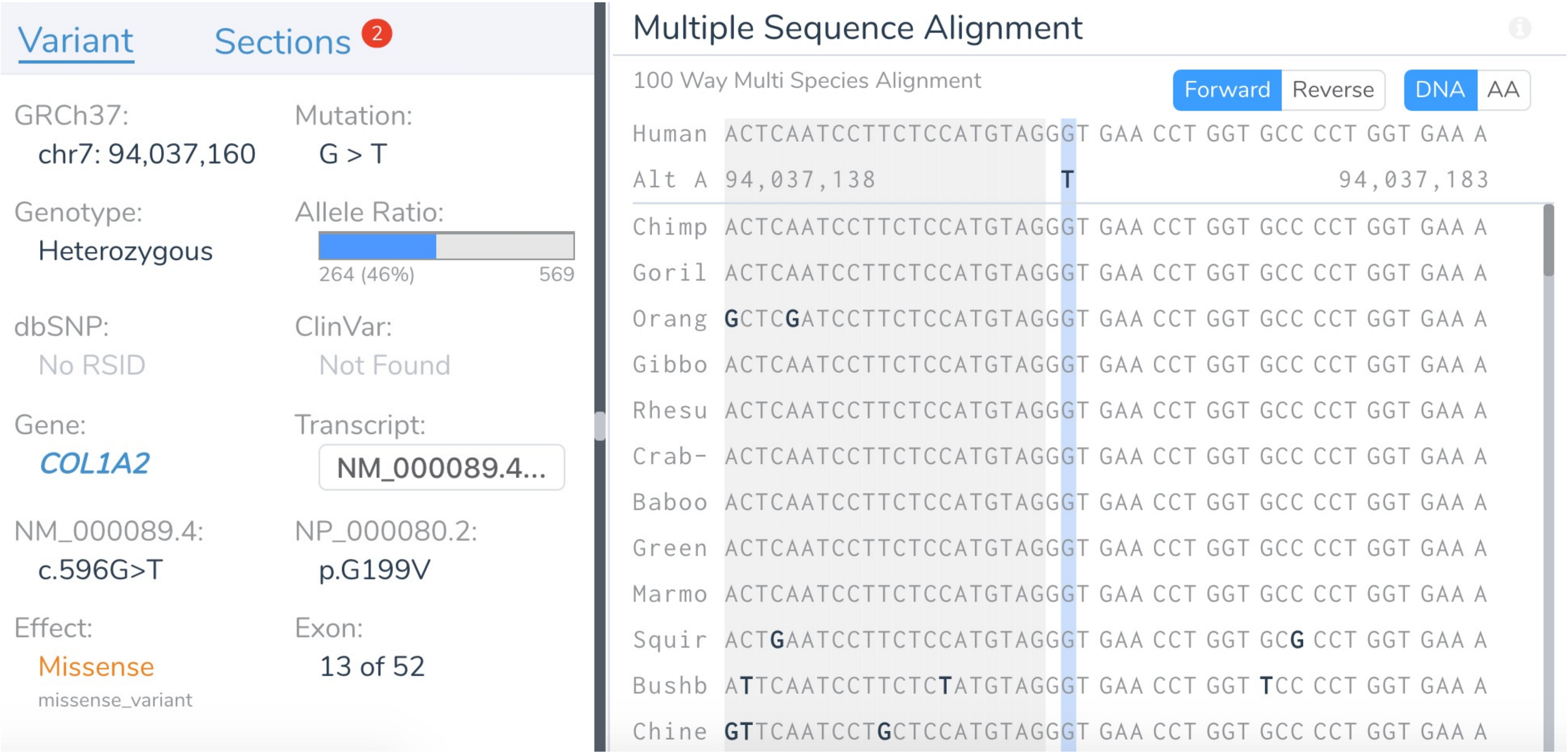
Variant
Sections 2
GRCh37:
chr7: 94,037,160
Mutation:
G > T
Genotype:
Heterozygous
Allele Ratio:
264 (46%)
569
dbSNP:
No RSID
ClinVar:
Not Found
Gene:
COL1A2
Transcript:
NM_000089.4...
NM_000089.4:
c.596G>T
NP_000080.2:
p.G199V
Effect:
Missense
missense_variant
Exon:
13 of 52
Multiple Sequence Alignment
100 Way Multi Species Alignment
Forward
Reverse
DNA
AA
Human ACTCAATCCTTCTCCATGTAGGGT GAA CCT GGT GCC CCT GGT GAA A
Alt A 94,037,138 T 94,037,183
Chimp ACTCAATCCTTCTCCATGTAGGGT GAA CCT GGT GCC CCT GGT GAA A
Goril ACTCAATCCTTCTCCATGTAGGGT GAA CCT GGT GCC CCT GGT GAA A
Orang GCTCGATCCTTCTCCATGTAGGGT GAA CCT GGT GCC CCT GGT GAA A
Gibbo ACTCAATCCTTCTCCATGTAGGGT GAA CCT GGT GCC CCT GGT GAA A
Rhesu ACTCAATCCTTCTCCATGTAGGGT GAA CCT GGT GCC CCT GGT GAA A
Crab- ACTCAATCCTTCTCCATGTAGGGT GAA CCT GGT GCC CCT GGT GAA A
Baboo ACTCAATCCTTCTCCATGTAGGGT GAA CCT GGT GCC CCT GGT GAA A
Green ACTCAATCCTTCTCCATGTAGGGT GAA CCT GGT GCC CCT GGT GAA A
Marmo ACTCAATCCTTCTCCATGTAGGGT GAA CCT GGT GCC CCT GGT GAA A
Squir ACTGAATCCTTCTCCATGTAGGGT GAA CCT GGT GCG CCT GGT GAA A
Bushb ATTCAATCCTTCTCTATGTAGGGT GAA CCT GGT TCC CCT GGT GAA A
Chine GTTCAATCCTGCTCCATGTAGGGT GAA CCT GGT GCC CCT GGT GAA A
