# Supplementary material for: Expanding the genetic and clinical spectrum of osteogenesis imperfecta: identification of novel rare pathogenic variants in type I collagen-encoding genes
Source: Front Endocrinol (Lausanne). 2023 Oct 20;14:1254695. doi: 10.3389/fendo.2023.1254695 (PMC10623311; doi:10.3389/fendo.2023.1254695)

Patient 21/21

<u>Chr17:g.48271303C>T</u>
<u>COL1A1(NM_000088.4) c.1767+1G>A</u>

chr17:48,271,303

Total count: 672
A : 0
C : 362 (54%, 362+, 0-)
G : 1 (0%, 1+, 0-)
T : 309 (46%, 309+, 0-)
N : 0

IGV Collapsed View

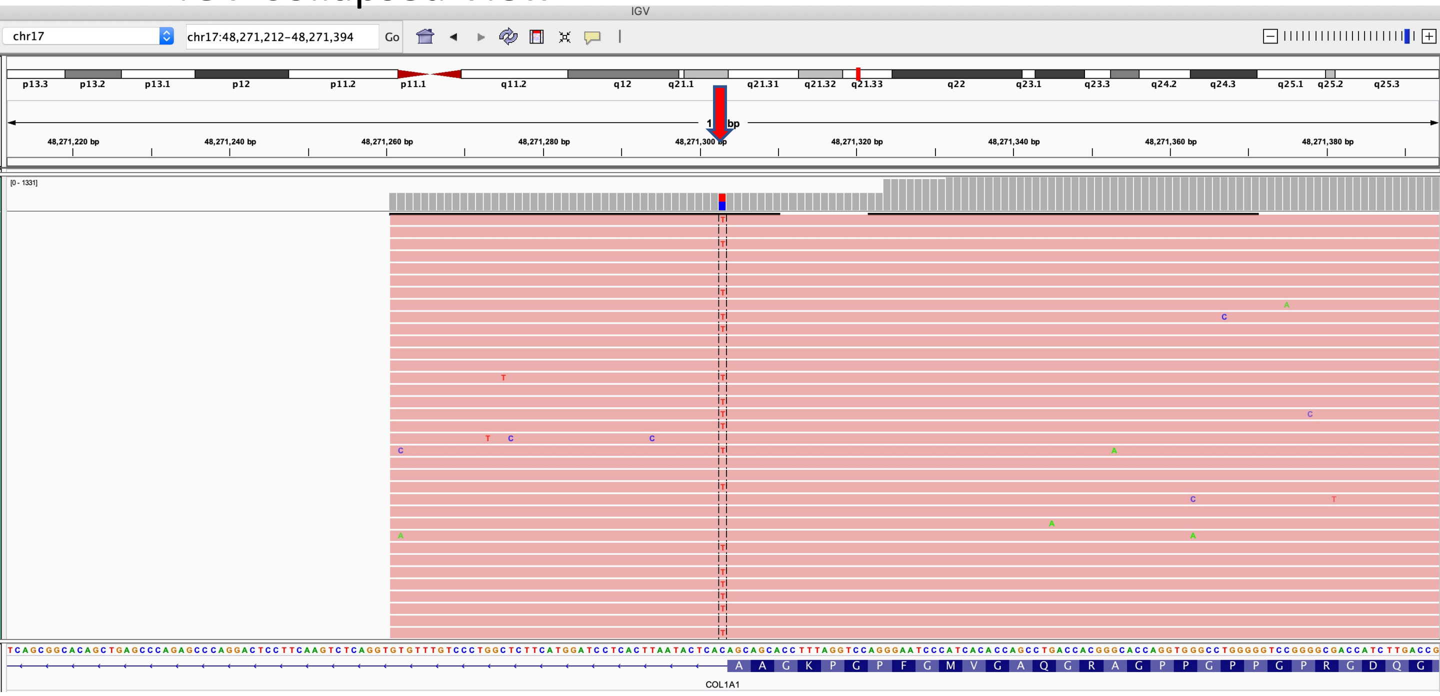

IGV Squished views

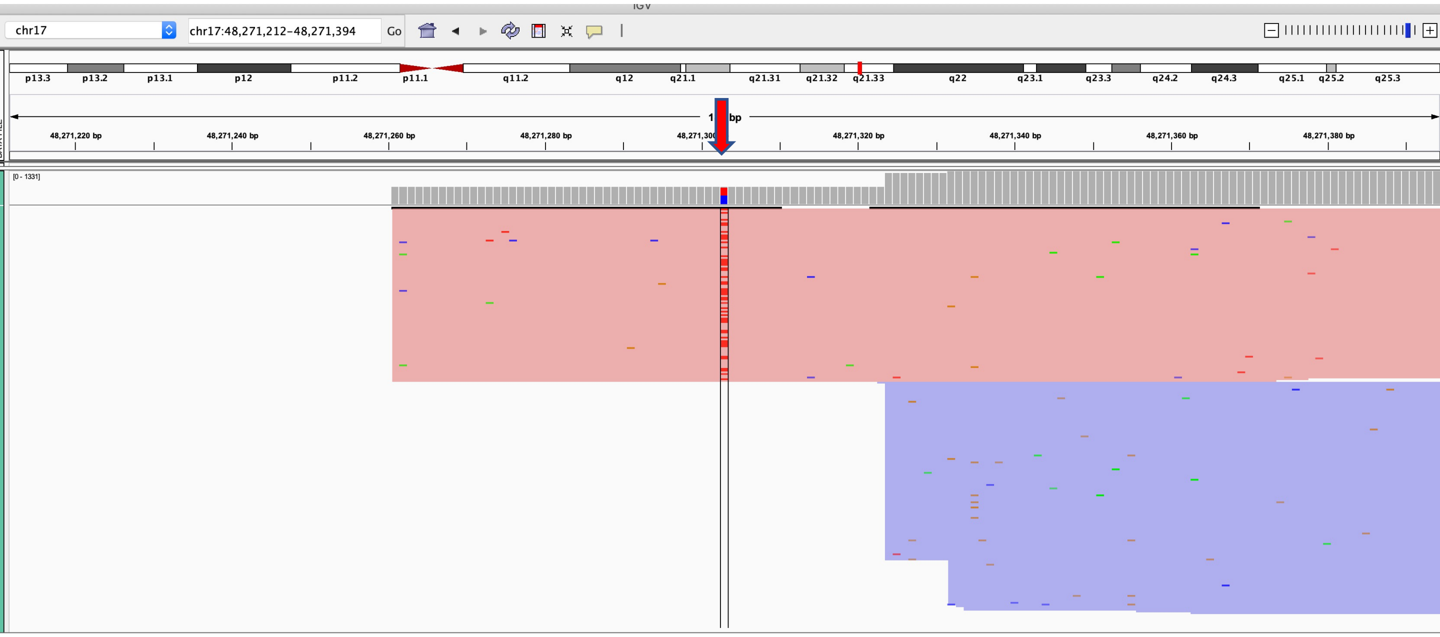

Patient 22/21

Chr17:g.48275131G>A
COL1A1(NM_000088.4):c.658C>T(p.Arg220Ter)

chr17:48,275,131

Total count: 1695
A : 885 (52%, 448+, 437-)
C : 1 (0%, 0+, 1-)
G : 809 (48%, 403+, 406-)
T : 0
N : 0

IGV Collapsed View

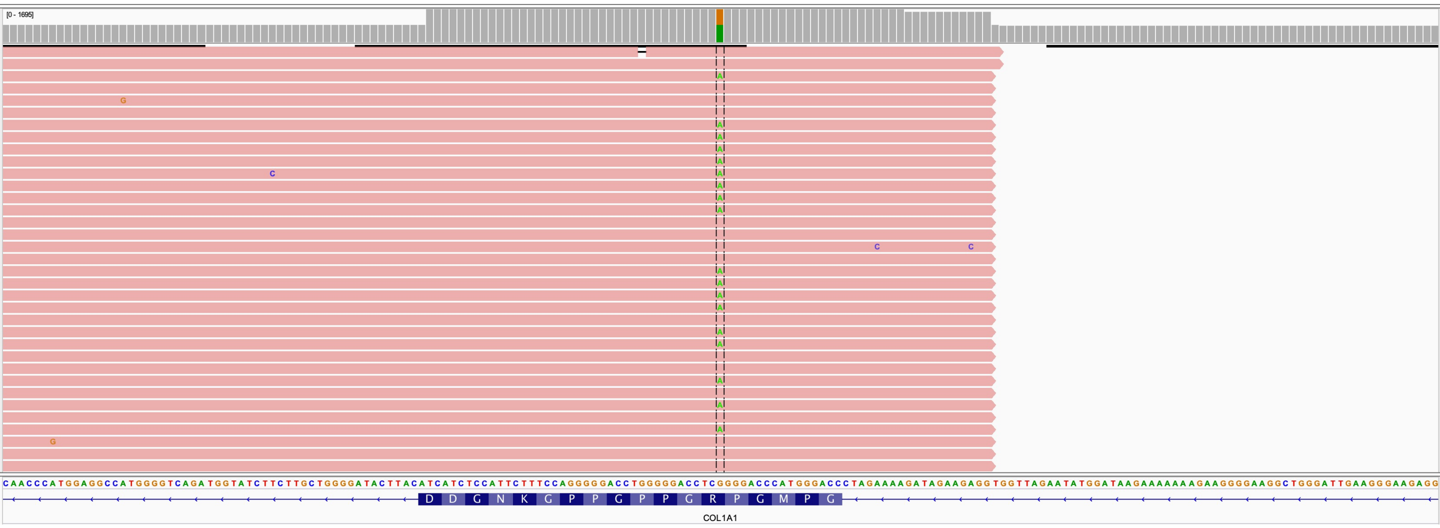

IGV Squished views

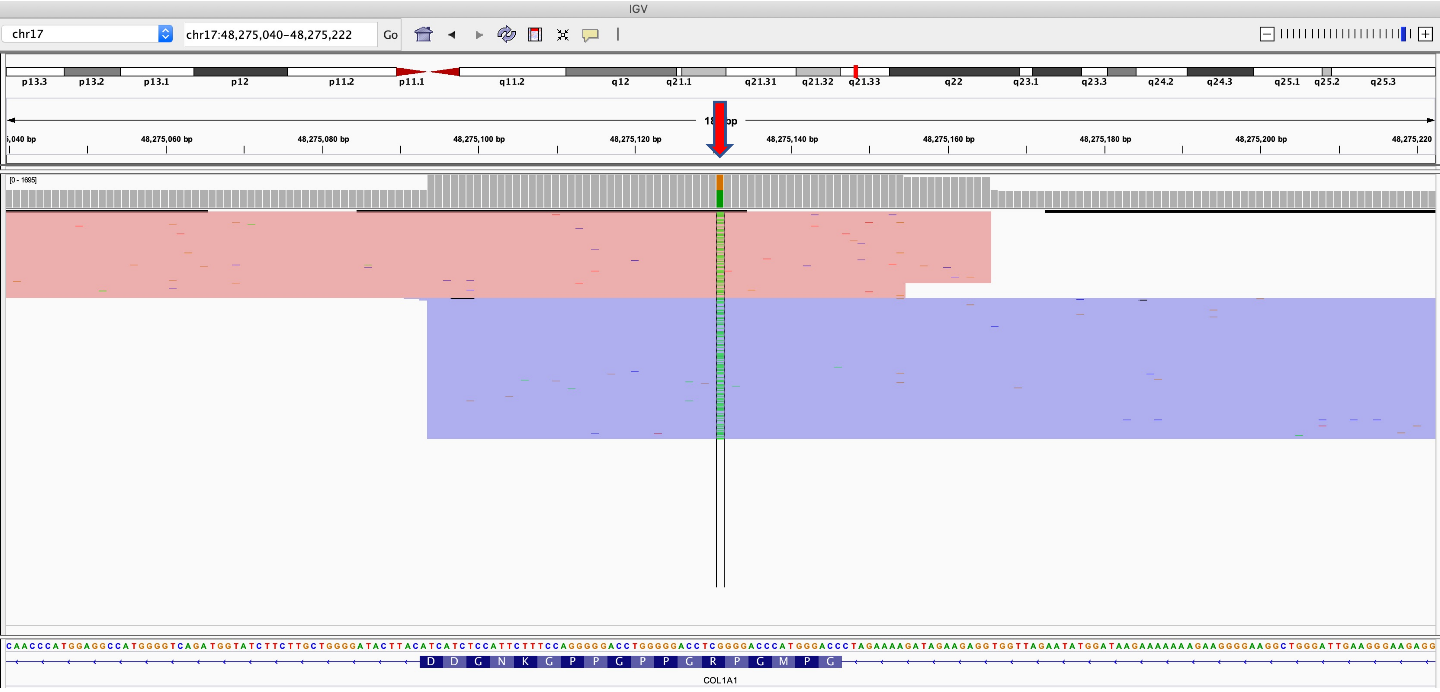

Patient 23/21

Chr17:g.48275131G>A
COL1A1(NM_000088.4):c.658C>T(p.Arg220Ter)

chr17:48,275,131

Total count: 2336
A : 1186 (51%, 591+, 595-)
C : 0
G : 1149 (49%, 571+, 578-)
T : 1 (0%, 1+, 0-)
N : 0

IGV Collapsed View

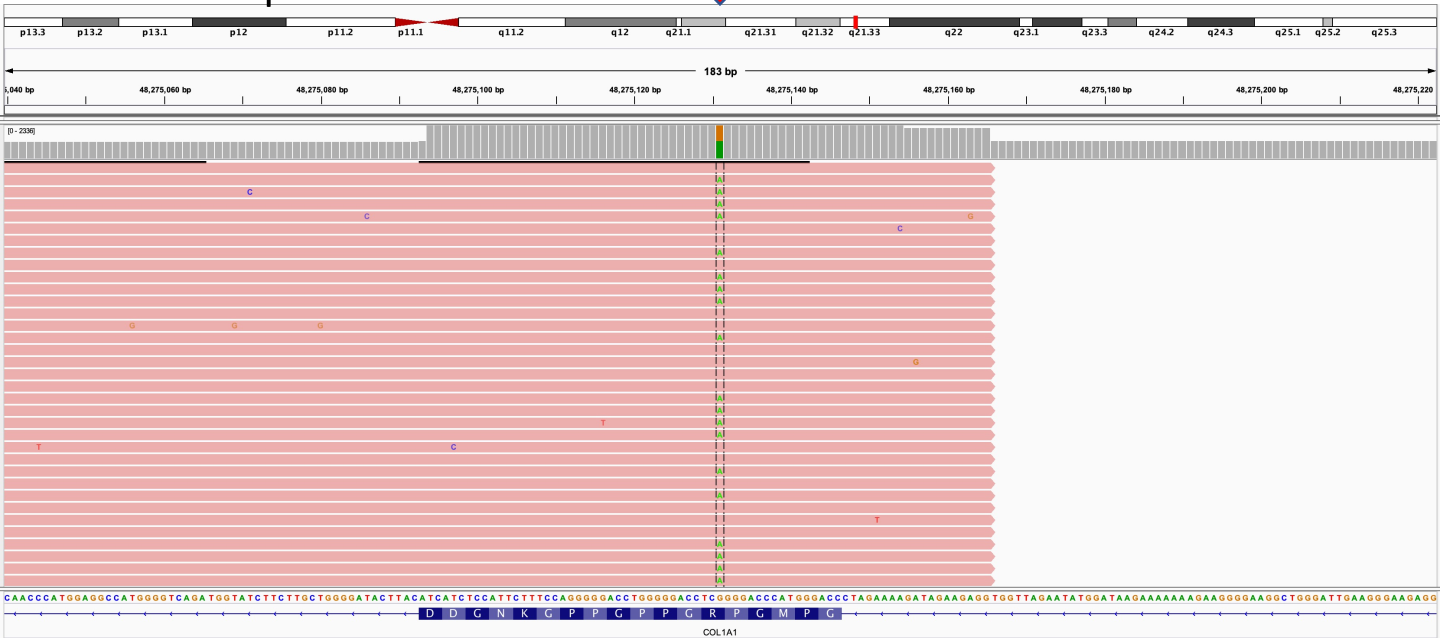

IGV Squished views

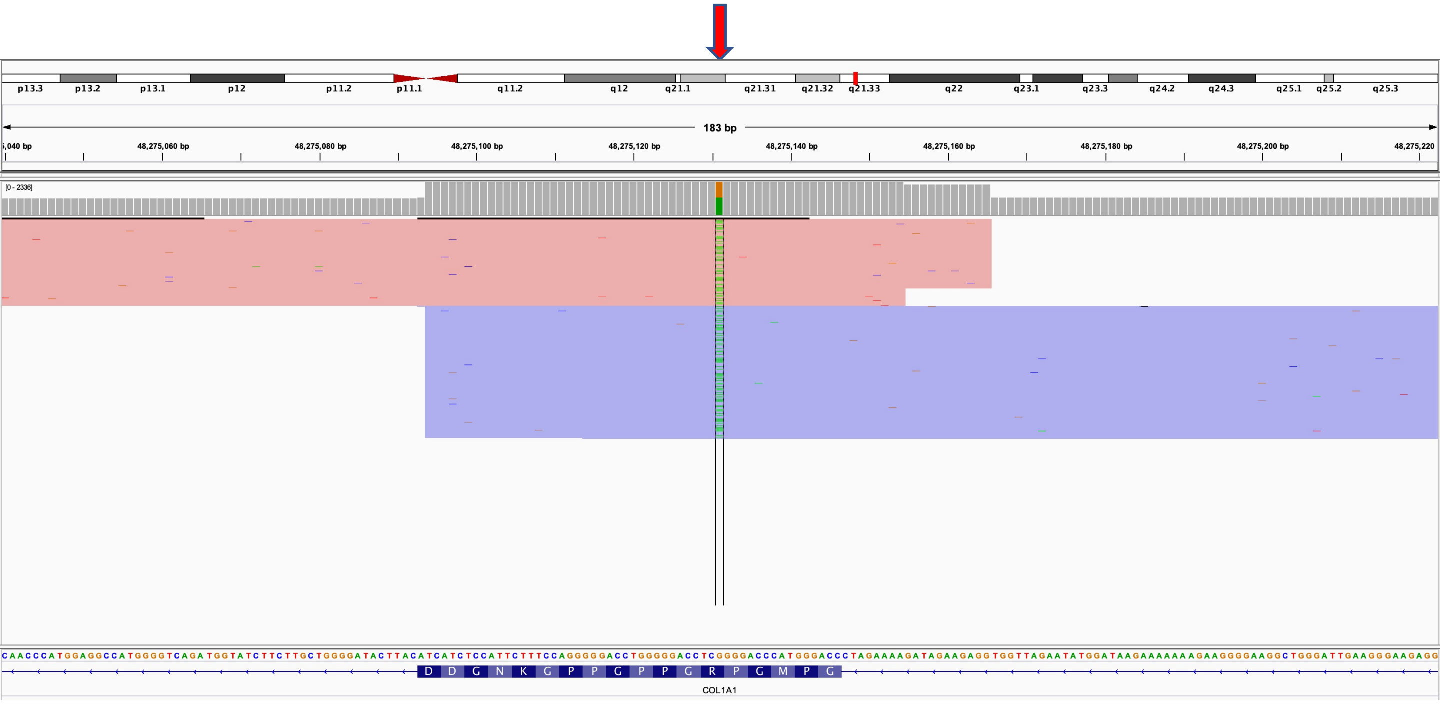

Patient 24/21

Chr17: g. 48266573delCAGG
COL1A1(NM_000088.4):c.2890_2893del (p.Pro964ValfsTer143)

chr17:48,266,573

Total count: 766
A : 3 (0%, 1+, 2-)
C : 763 (100%, 442+, 321-)
G : 0
T : 0
N : 0

DEL: 373
INS: 0

chr17:48,266,574

Total count: 766
A : 764 (100%, 442+, 322-)
C : 0
G : 2 (0%, 1+, 1-)
T : 0
N : 0

DEL: 373
INS: 0

chr17:48,266,575

Total count: 768
A : 0
C : 0
G : 768 (100%, 444+, 324-)
T : 0
N : 0

DEL: 372
INS: 0

chr17:48,266,576

Total count: 768
A : 0
C : 0
G : 767 (100%, 443+, 324-)
T : 1 (0%, 1+, 0-)
N : 0

DEL: 372
INS: 0

IGV Collapsed View

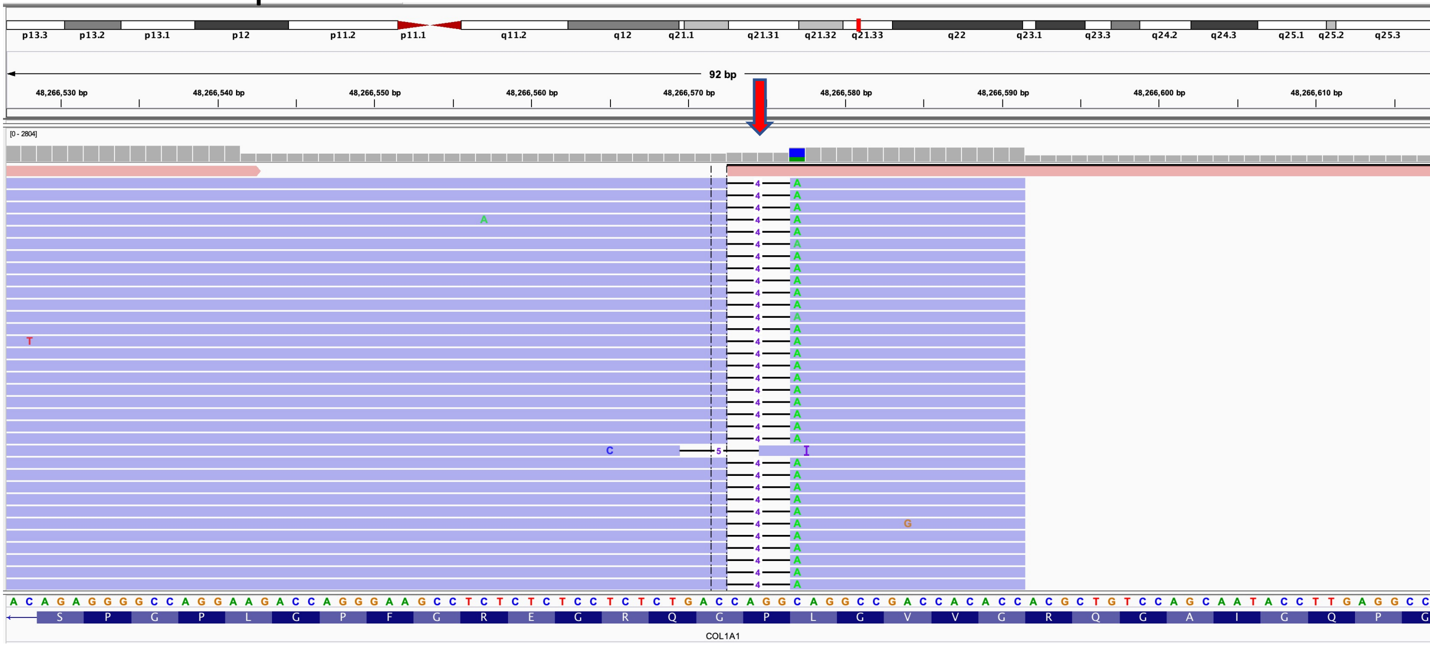

IGV Squished views

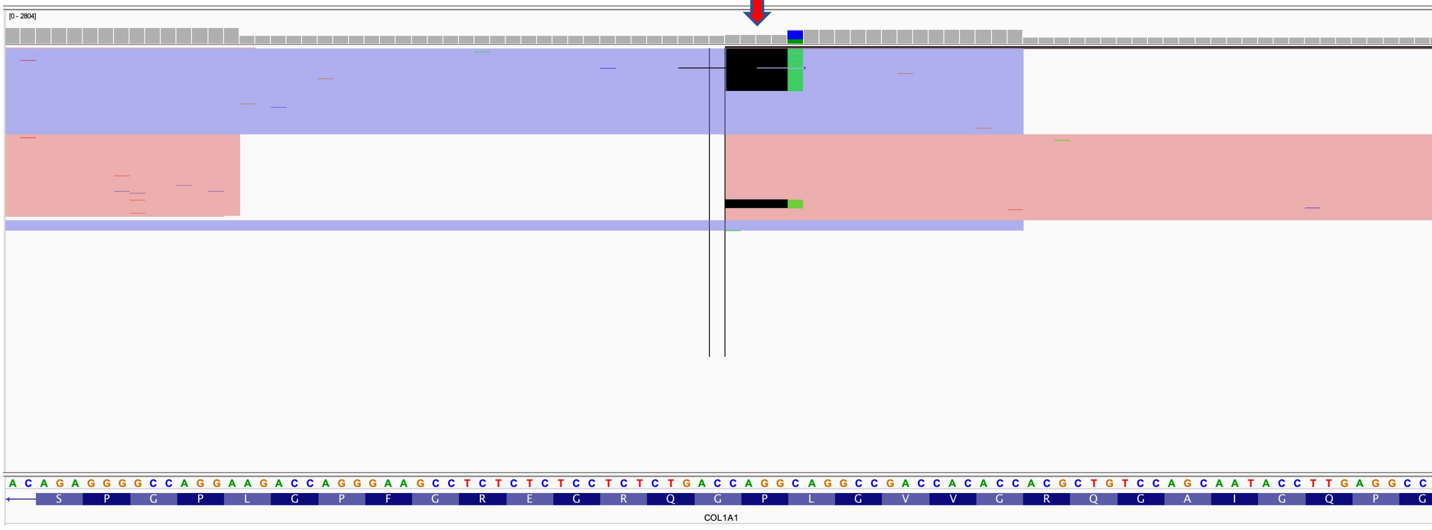

Patient 25/21

Chr17: g.48266573delCAGG
COL1A1(NM_000088.4):c.2890_2893del (p.Pro964ValfsTer143)

chr17:48,266,573
Total count: 717
A : 1 (0%, 1+, 0-)
C : 716 (100%, 430+, 286-)
G : 0
T : 0
N : 0

DEL: 321
INS: 1

chr17:48,266,574
Total count: 716
A : 715 (100%, 430+, 285-)
C : 0
G : 0
T : 1 (0%, 0+, 1-)
N : 0

DEL: 322
INS: 0

chr17:48,266,575
Total count: 717
A : 1 (0%, 1+, 0-)
C : 0
G : 716 (100%, 429+, 287-)
T : 0
N : 0

DEL: 321
INS: 0

chr17:48,266,576
Total count: 715
A : 0
C : 0
G : 715 (100%, 430+, 285-)
T : 0
N : 0

DEL: 323
INS: 0

IGV Collapsed View

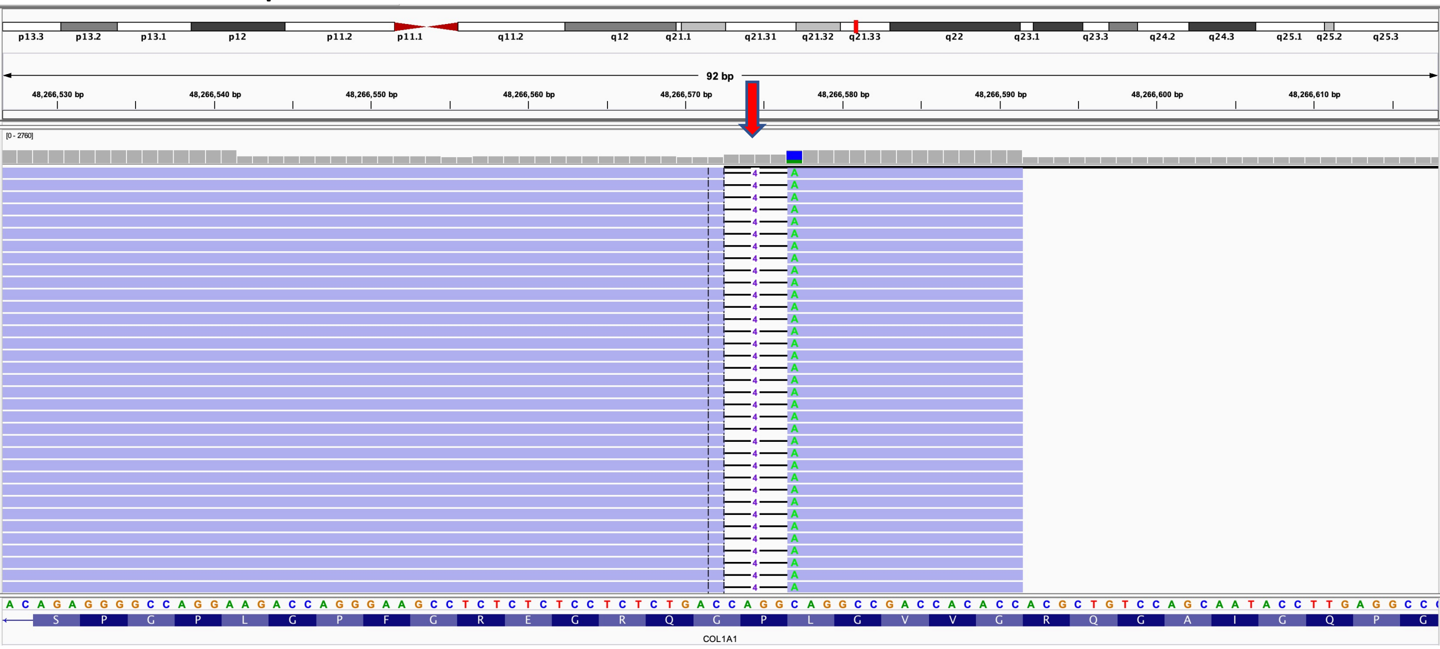

IGV Squished views

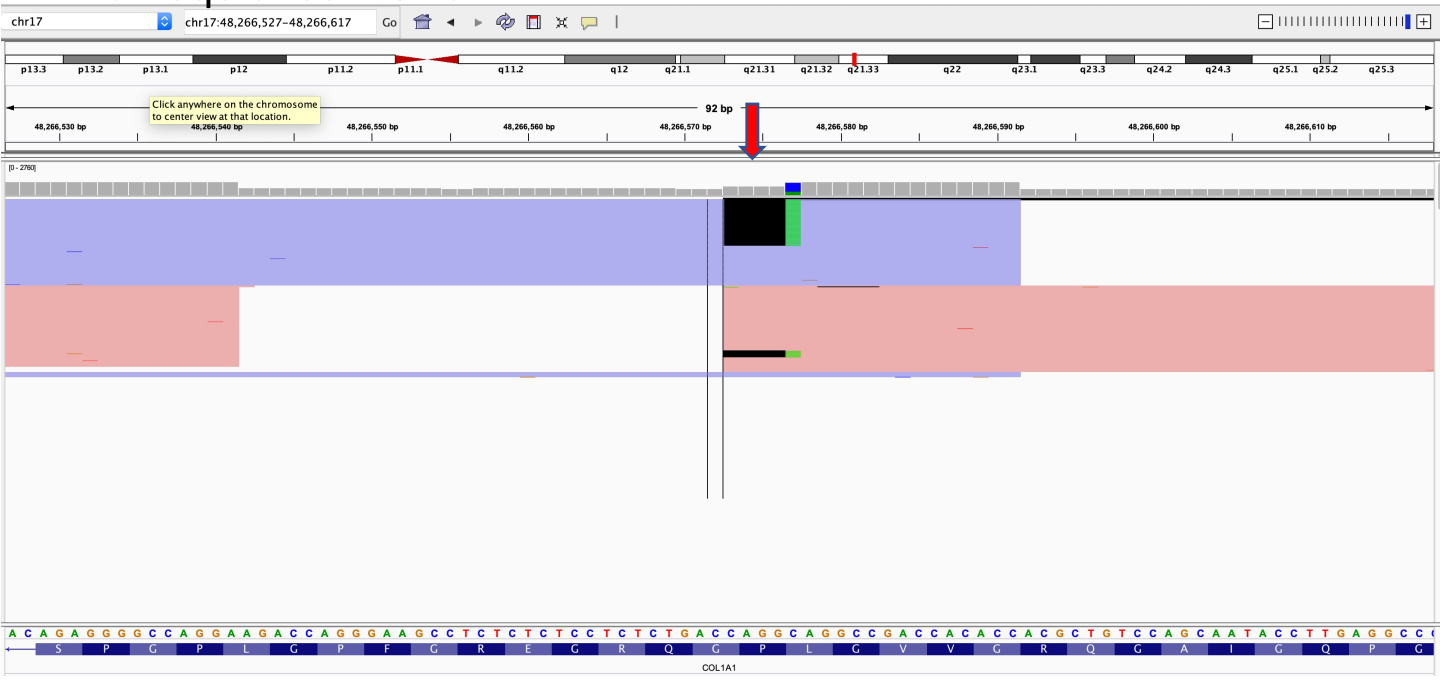

Patient 01/22

Chr17:g.48275820C>A

COL1A1(NM_000088.4): c.517G>T(p.Gly173Ter)

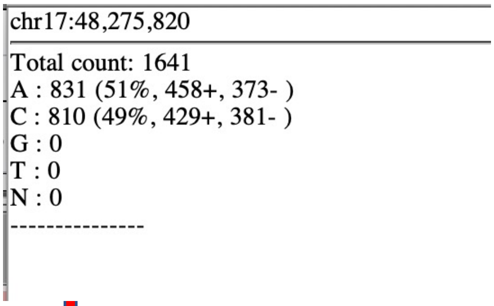

IGV Collapsed View

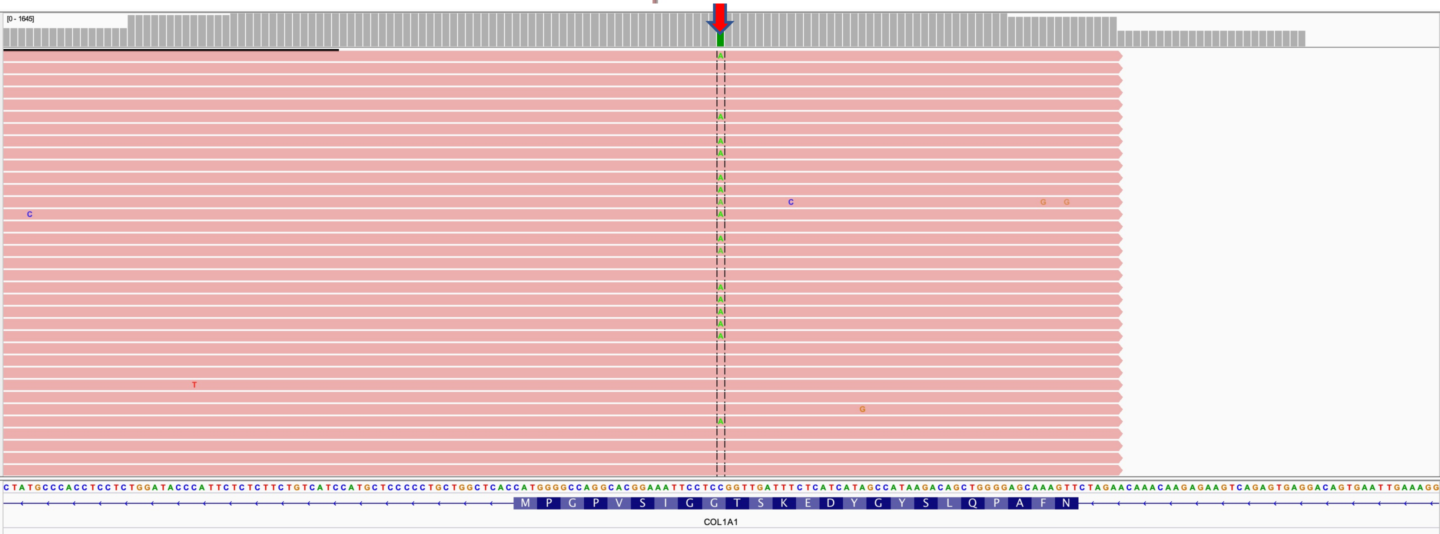

IGV quished views

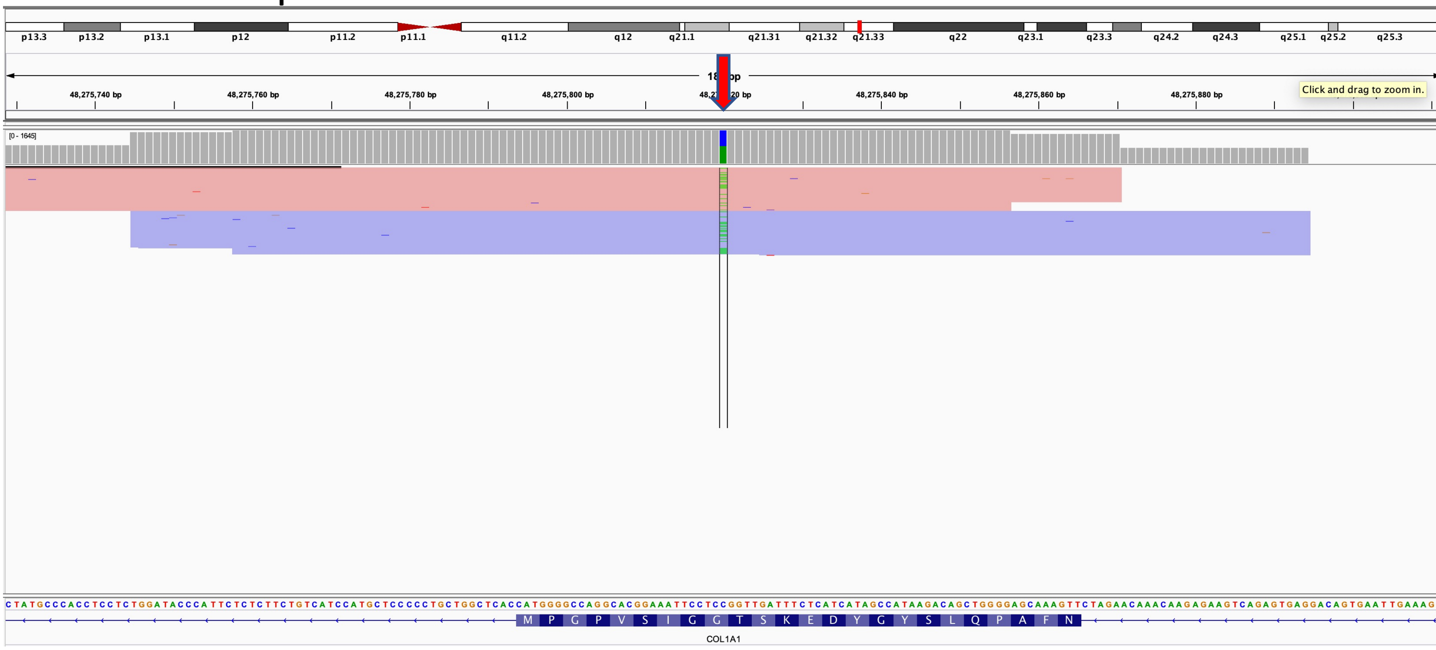

Patient 02/22

Chr17:g.48263796delC
COL1A1(NM_000088.4):c.3887del (p.Gly1296ValfsTer35)

chr17:48,263,796

Total count: 917
A : 1 (0%, 0+, 1-)
C : 914 (100%, 457+, 457-)
G : 2 (0%, 1+, 1-)
T : 0
N : 0

DEL: 903
INS: 0

IGV Collapsed View

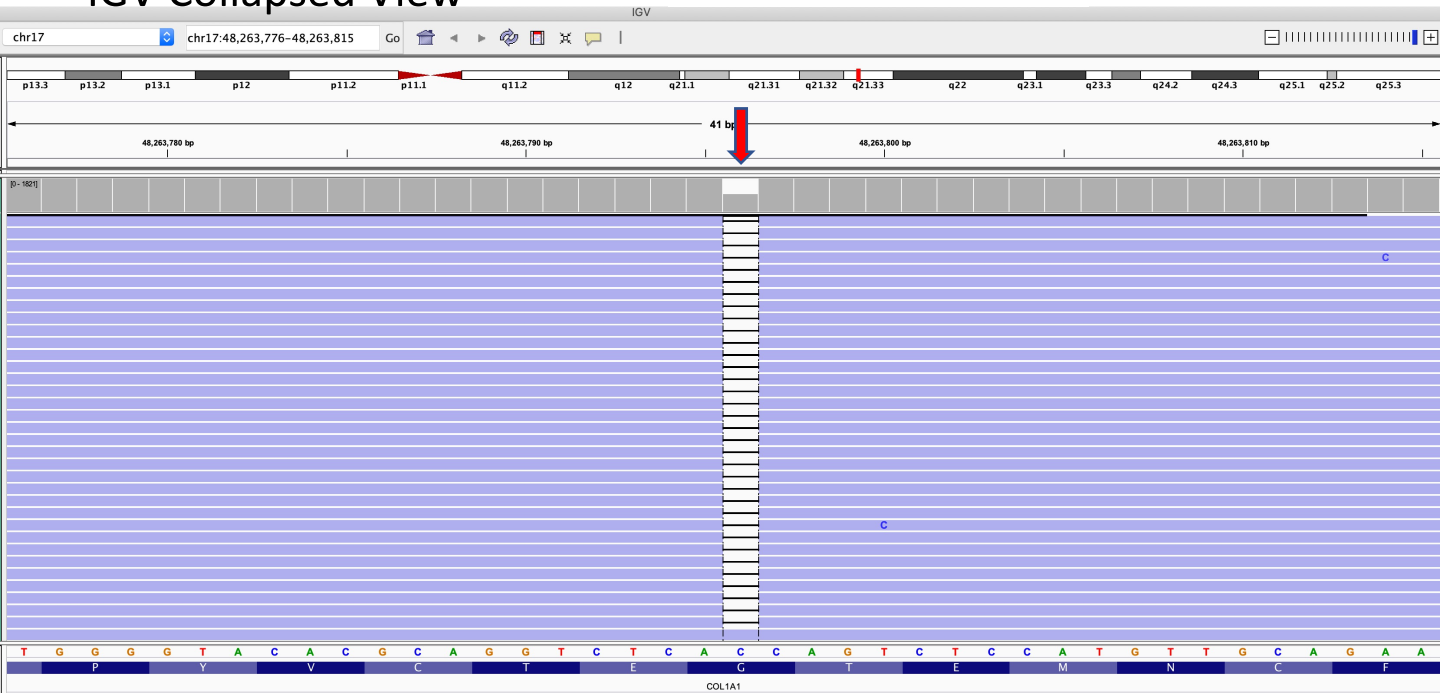

IGV Squished views

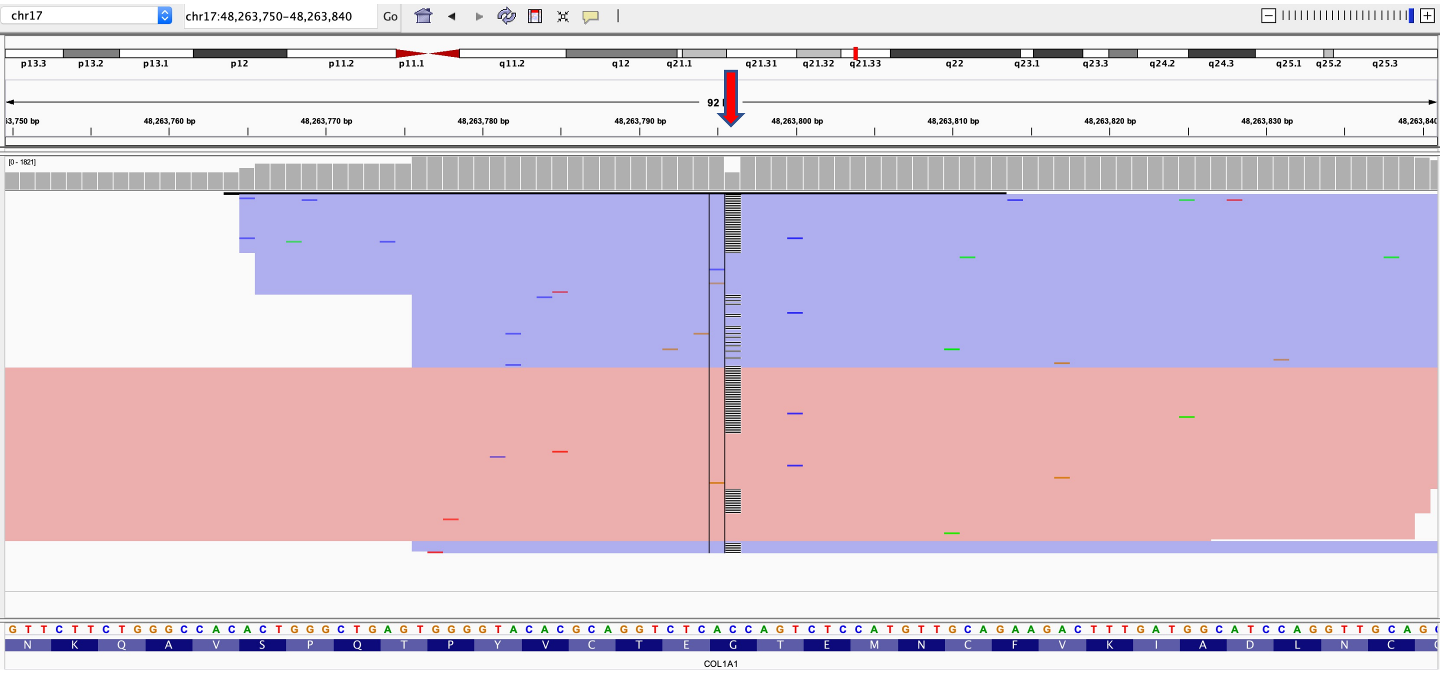

Patient 04/22

Chr7:g.94039107G>A
COL1A2(NM_000089.4):c.1009G>A (p.Gly337Ser)

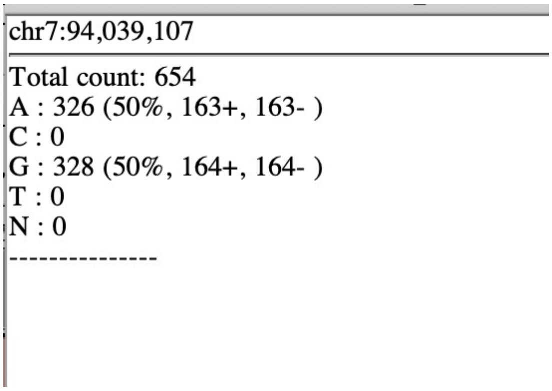

IGV Collapsed View

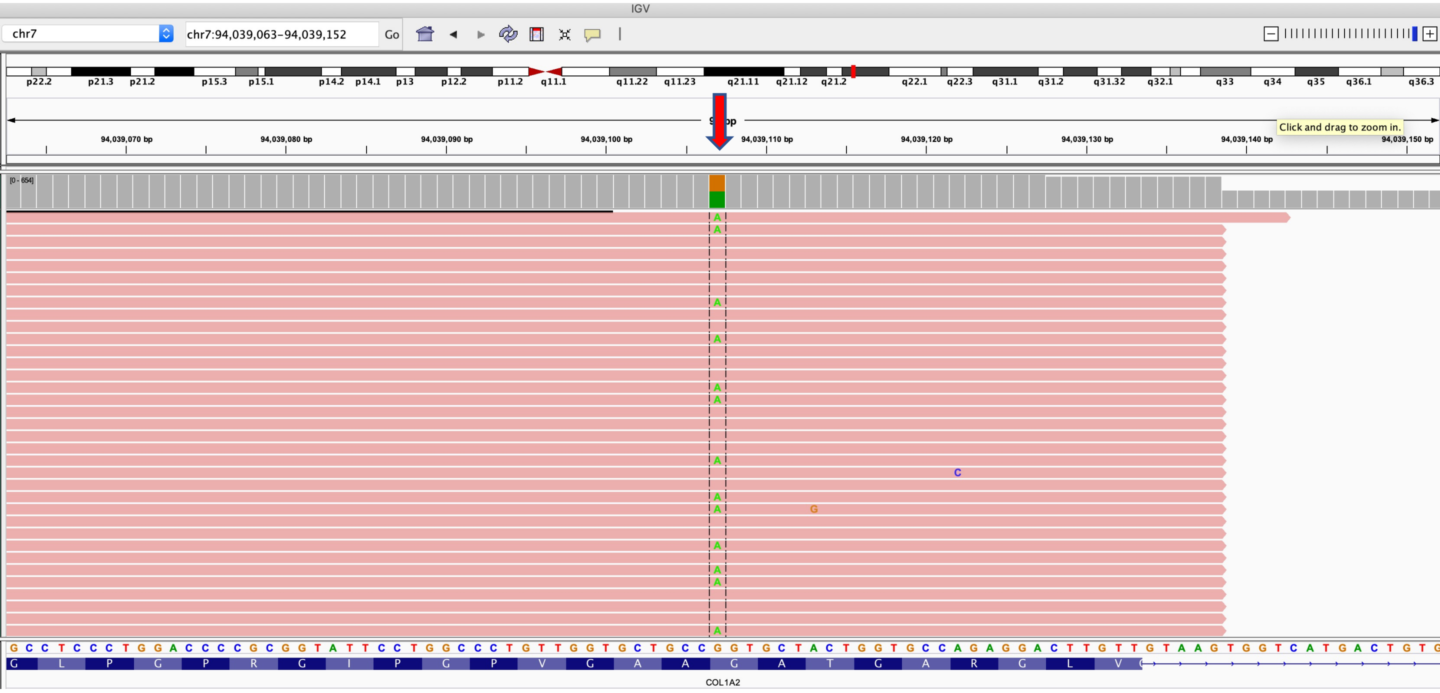

IGV Squished views

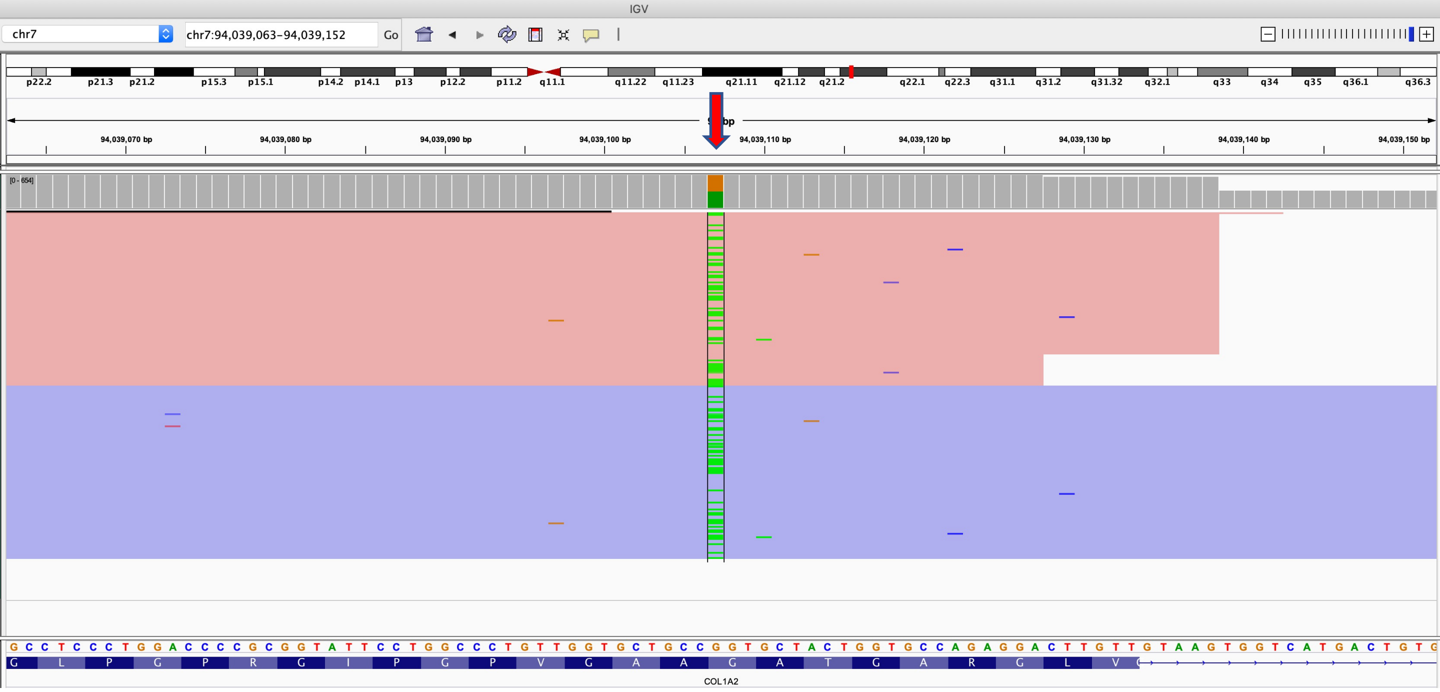

Patient 05/22

Chr7:g.94037160G>T

COL1A2(NM_000089.4):c.596G>T (p.Gly199Val)

chr7:94,037,160

Total count: 1440
A : 0
C : 0
G : 714 (50%, 357+, 357-)
T : 726 (50%, 360+, 366-)
N : 0

IGV Collapsed View

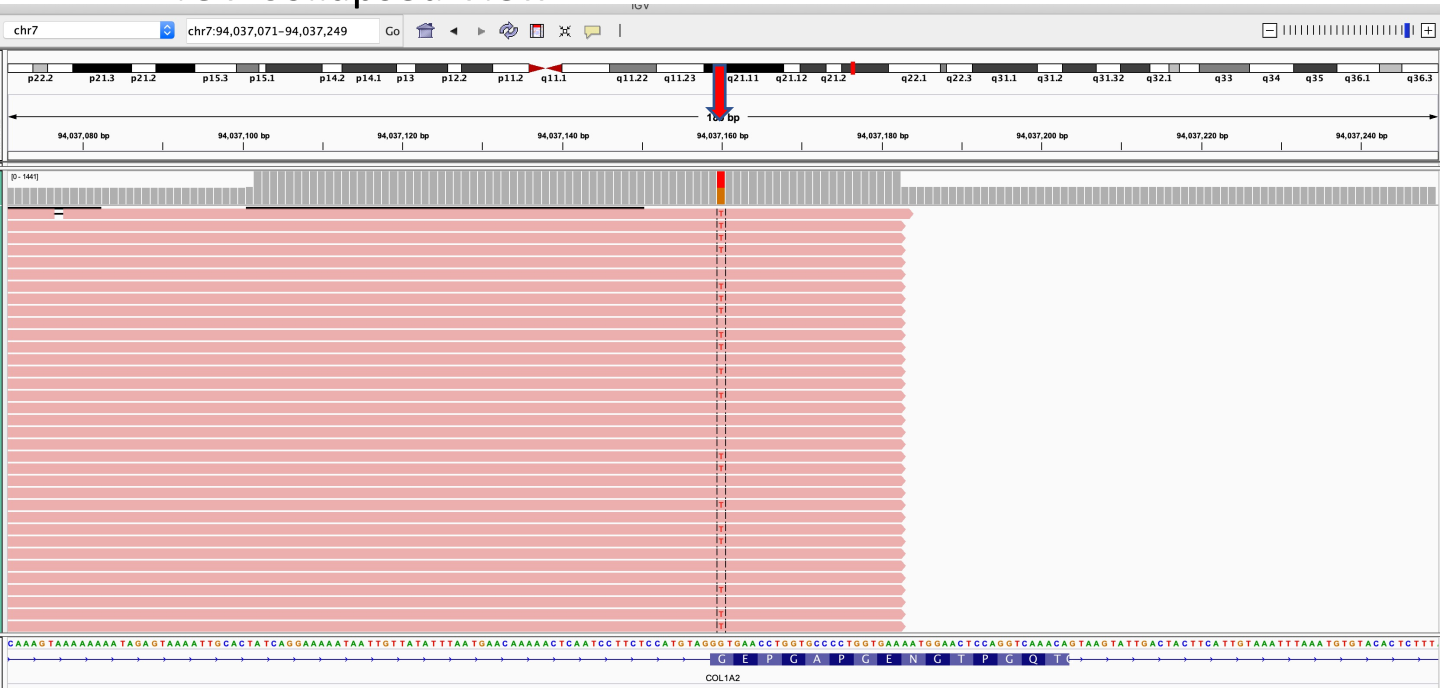

IGV quished views

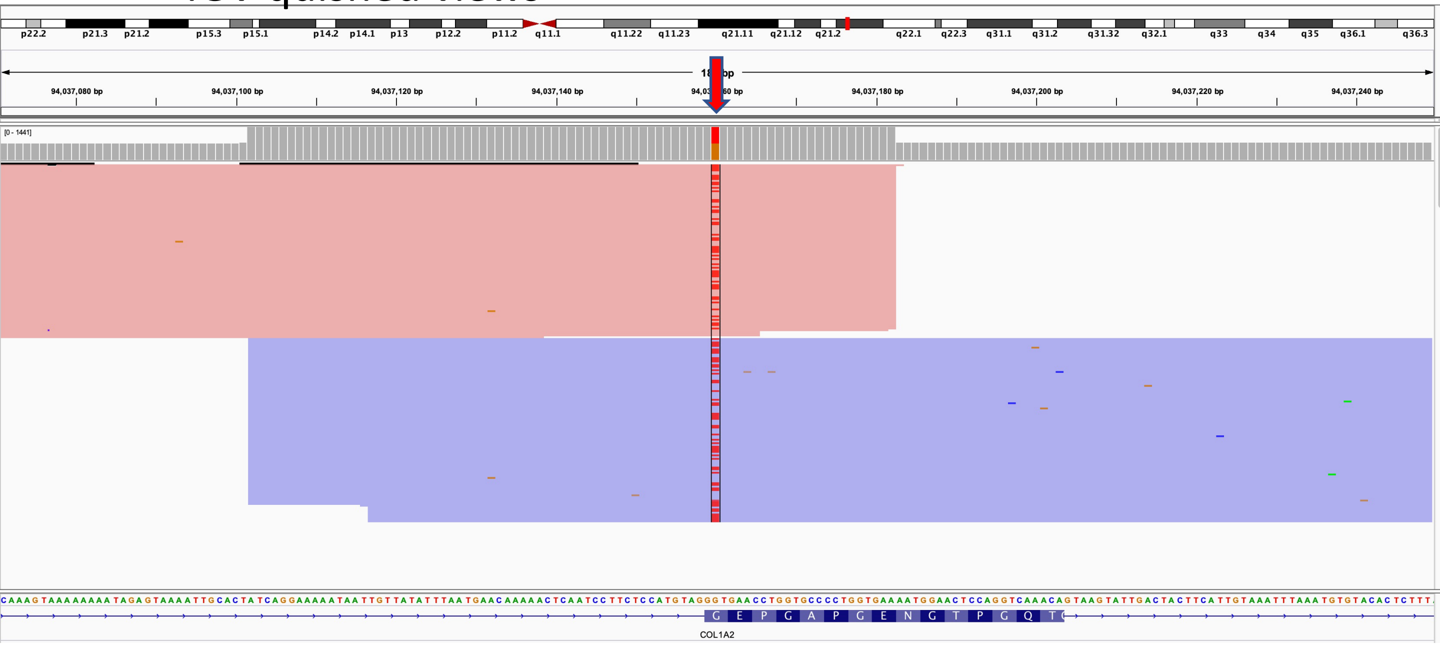

Patient 06/22

Chr7:g.94037160G>T

COL1A2(NM_000089.4):c.596G>T (p.Gly199Val)

chr7:94,037,163

Total count: 1129
A : 1127 (100%, 560+, 567-)
C : 0
G : 2 (0%, 1+, 1-)
T : 0
N : 0

IGV Collapsed View

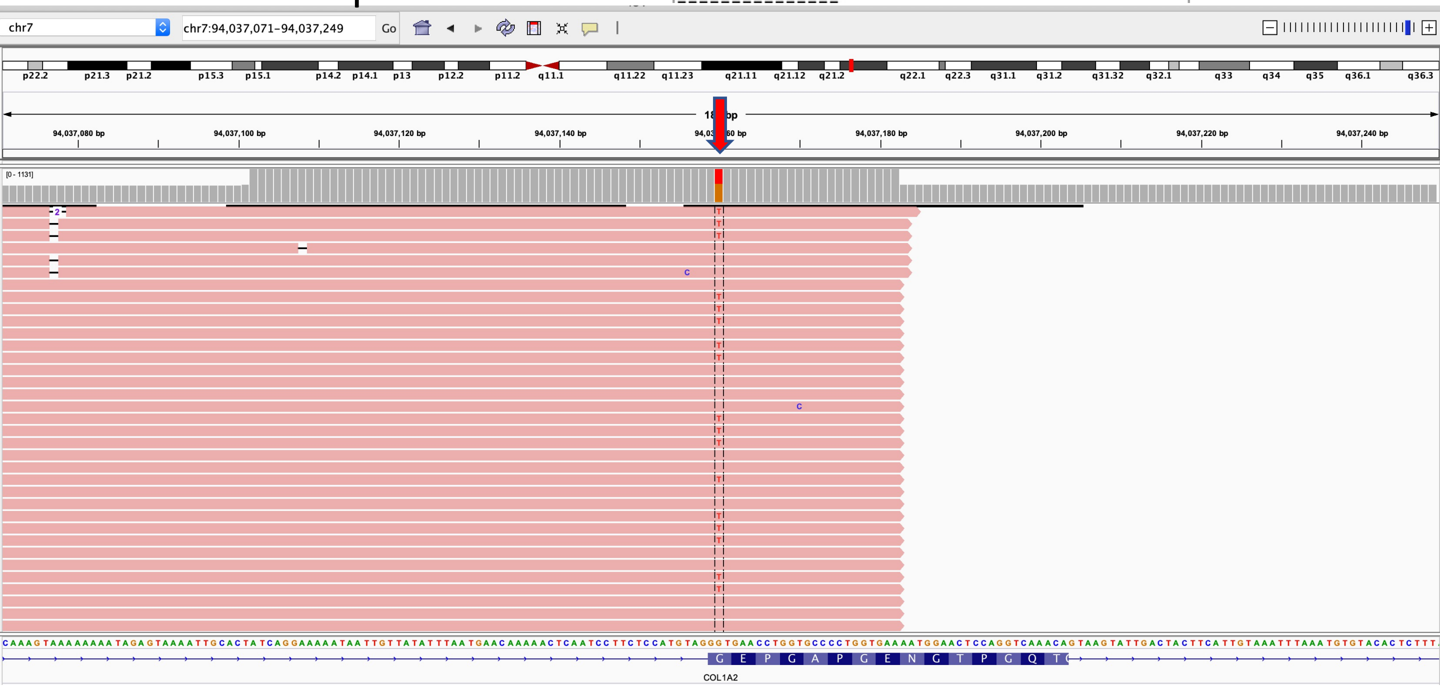

IGV quished views

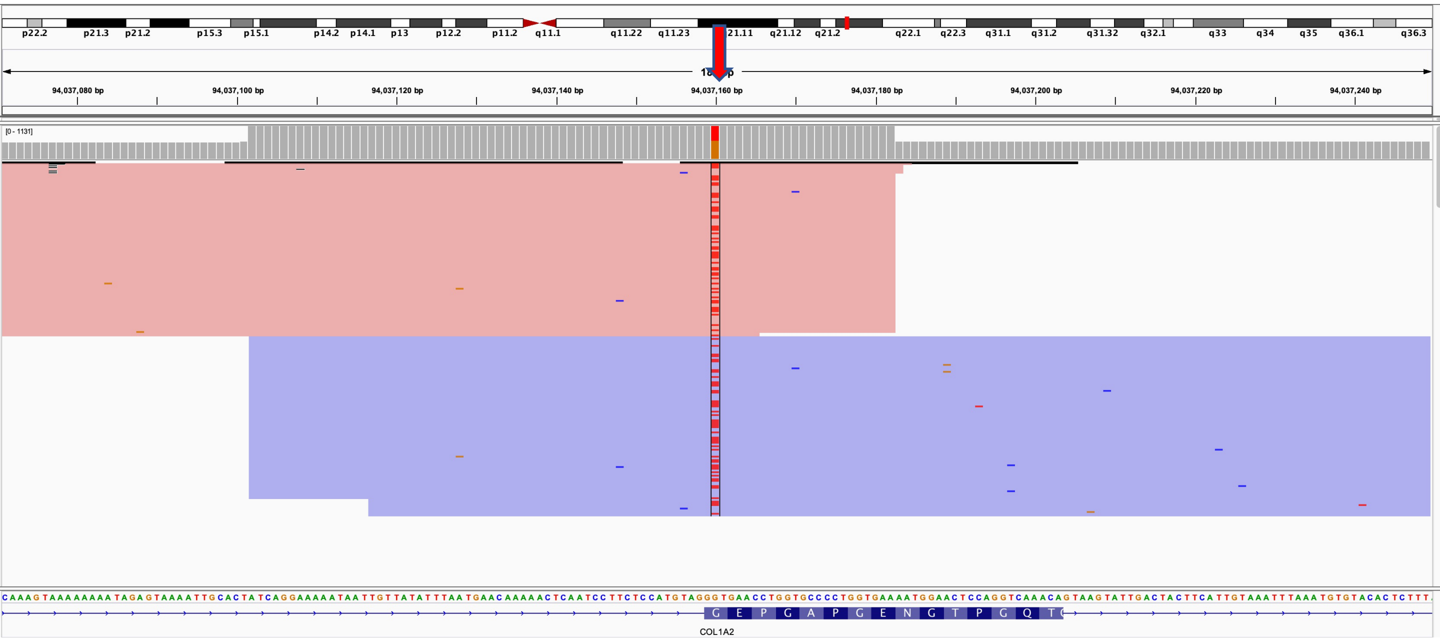

Supplement: Supplementary file 5 [file Image_5.pdf]
